# Polyelectrolyte Complex/MMT Hybrid for SPI/WE Adhesives with Balanced Flame Retardancy and Shear Strength

**DOI:** 10.3390/gels12090834

**Published:** 2026-09-11

**Authors:** Han Yang, Yuxin Luo, Shiyi Ding, Chunchun Wu, Tao Zhang

**Affiliations:** 1 School of Materials Science and Engineering, Zhejiang University, Hangzhou 310058, China; wuchun@zju.edu.cn; 2 Zhejiang Provincial Fire and Rescue Bureau, Hangzhou 310008, China; srsrsre@163.com; 3 School of Materials Science & Engineering, Zhejiang Sci-Tech University, Hangzhou 310018, China; yamy_luo@163.com; 4 Department of Product Design, School of Art & Design, Zhejiang Sci-Tech University, Hangzhou 310018, China

**Keywords:** soybean protein isolate, waterborne epoxy, polyelectrolyte complex, plywood, flame retardancy, shear strength

## Abstract

Soybean protein isolate (SPI) adhesives are eco-friendly but suffer from poor water resistance and bonding strength. Waterborne epoxy (WE) can effectively improve these properties; however, WE-modified SPI adhesives can hardly satisfy the growing stringent flame-retardant demands for plywood. Herein, we report a composite adhesive (SPI/20WE/THPS@CS/MMT) that simultaneously exhibits favorable flame retardancy and dry and wet shear strengths. In this system, a phosphorus-, nitrogen-, and sulfur-containing polyelectrolyte complex (THPS@CS) is fabricated by vacuum-drying an ionically crosslinked polyelectrolyte hydrogel derived from bis[tetrakis(hydroxymethyl)phosphonium] sulfate (THPS) and chitosan (CS) and subsequently incorporated into the SPI/WE matrix (SPI/20WE) together with montmorillonite (MMT). The plywood bonded with SPI/20WE/THPS@CS/MMT achieves a limiting oxygen index (LOI) of 28.0% and shows significantly reduced heat release in cone calorimeter tests. Meanwhile, the resultant plywood possesses high dry and wet shear strengths that fulfill the interior-use plywood criteria specified in GB/T 9846–2015. This work offers new insights into the design and fabrication of high-performance eco-friendly plywood adhesives.

## 1. Introduction

The plywood industry has long relied on synthetic, petroleum-based adhesives, which deliver high bonding strength and favorable water resistance [1]. However, the production and application of these petroleum-based adhesives bring considerable environmental and health concerns, owing to their dependence on non-renewable resources and formaldehyde emissions [2]. To address these challenges, developing bio-based adhesives derived from renewable biomass has become a viable strategy to reduce the consumption of petroleum-based adhesives in the plywood industry, as they possess distinct advantages, including renewable raw-material sources, biodegradability, and low toxic-substance release [3]. Among the bio-based adhesives, soybean protein isolate (SPI) has drawn extensive attention as a promising bio-based raw material for wood adhesives owing to its abundant natural resources, low cost, biodegradability, and formaldehyde-free feature, which conforms to the sustainable development trend of the plywood industry [4]. However, SPI contains a large number of hydrophilic groups such as amino, hydroxyl, and carboxyl, and its internal network primarily relies on weak intermolecular hydrogen bonds [5]. Consequently, the adhesive easily absorbs water, swells, and undergoes chain dissociation under humid conditions, leading to a drastic decline in interfacial bonding strength [6]. This poor water resistance and the resulting low wet shear strength remain the major obstacles limiting the practical application of SPI adhesives in plywood.

To overcome the above drawbacks, various strategies have been explored, including chemical crosslinking, physical blending, and protein molecular modification [7]. Among them, waterborne epoxy (WE) stands out as an effective modifier because it uses water as a dispersion medium and has low volatile organic compound (VOC) emissions [8,9]. Previous studies have shown that WE has high crosslinking reactivity with amino and hydroxyl groups of SPI to form a dense chemical crosslinking network, effectively blocking hydrophilic sites, thereby improving the water resistance and bonding strength of the resulting adhesive [10,11]. In principle, owing to its reactivity toward amino and hydroxyl groups, WE holds great potential for modifying natural protein-based adhesives. However, with increasing public awareness of fire safety for construction [12], higher requirements have been raised for the flame retardancy of wood adhesives. Unfortunately, pristine WE is inherently flammable [13]. The flame retardancy of WE-modified SPI adhesives (SPI/WE) can hardly meet the increasingly stringent requirements. Hence, it is urgently required to endow SPI/WE with satisfactory flame retardancy while maintaining favorable bonding performance.

Current approaches to enhancing the flame retardancy of epoxy-containing adhesives fall into two categories [14]. One is intrinsic flame retardancy, where flame-retardant elements are covalently incorporated into the molecular chain. The other is additive flame retardancy, where flame-retardant additives are physically mixed into the matrix. Among them, the intumescent flame-retardant system has been recognized as one of the most promising candidates, benefiting from its low smoke, halogen-free feature and excellent condensed-phase charring effect [15]. However, two challenges still limit the further development of flame-retardant SPI/WE adhesives. On one hand, intrinsic flame retardancy can provide durable and efficient performance at the molecular level, but it usually requires complex chemical synthesis. On the other hand, additive flame retardancy is more convenient and industrially feasible. However, commercially available flame retardants that are compatible with SPI/WE systems and do not severely deteriorate bonding strength are still limited.

Polyelectrolyte hydrogels formed via electrostatic complexation of oppositely charged polyelectrolytes have attracted substantial interest owing to their facile preparation, tunable network structure, and multi-functional capabilities [16]. Inspired by these merits, this work fabricates a phosphorus-, nitrogen-, and sulfur-containing polyelectrolyte hydrogel from bis[tetrakis(hydroxymethyl)phosphonium] sulfate solution (THPS) and chitosan (CS). After vacuum-drying, the resultant solid polyelectrolyte complex (THPS@CS) is used as a flame-retardant additive for SPI/WE adhesive together with montmorillonite (MMT) nanosheets. As a quaternary phosphonium salt, THPS features a central phosphorus atom bonded to four hydroxymethyl groups, which can generate phosphorus-containing acids that catalyze char formation [17]. Meanwhile, its sulfate counterion can release inert gases (e.g., SO_2_) to dilute combustible volatiles. CS, a renewable carbon-rich polysaccharide, mainly acts as a carbon source, which can be dehydrated and carbonized under heating to form a protective char layer [18]. Moreover, its amino groups can release inert nitrogen-containing gases and can also serve as a gas source. The MMT is selected because it serves as a physical barrier to block the transfer of heat and oxygen, further enhancing the flame-retardant performance [19]. Beyond flame retardancy, the polar groups of THPS@CS can interact with the SPI/WE matrix, while the dispersed MMT functions as a reinforcing filler to enhance bonding strength and water resistance. The results demonstrate that plywood bonded with the obtained SPI/20WE/THPS@CS/MMT composite adhesive achieves a limiting oxygen index (LOI) of 28.0% and shows obviously reduced heat release in cone calorimeter tests. Moreover, the plywood demonstrates high dry and wet shear strengths, meeting the requirements of the standard for interior-use plywood specified in GB/T 9846–2015 [20], thus providing new insights into the design and fabrication of eco-friendly plywood adhesives.

## 2. Results and Discussion

### 2.1. Characterization of THPS@CS Polyelectrolyte Complex

**Figure 1:**
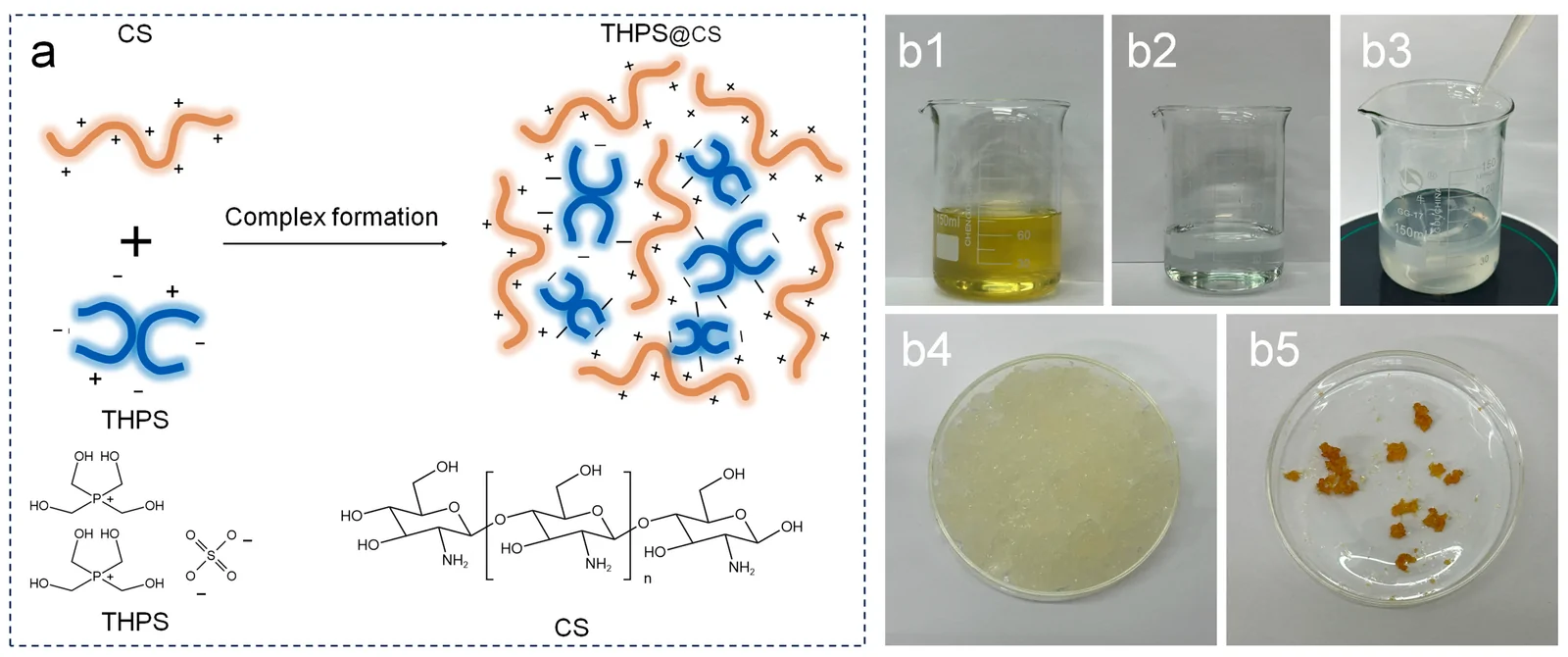
(**a**) Preparation illustration of THPS@CS polyelectrolyte complex. Photographs of the (**b1**) CS solution, (**b2**) THPS solution, (**b3**) formation of transparent polyelectrolyte hydrogel upon addition of CS solution into THPS solution, (**b4**) collected THPS@CS polyelectrolyte hydrogel before drying and (**b5**) solid THPS@CS polyelectrolyte complex after drying.

Figure 1 shows the preparation process of THPS@CS complex. The THPS solution is colorless and transparent, whereas the CS solution is light yellow. Adding the CS solution dropwise to the THPS solution yields a transparent hydrogel, which is vacuum-dried to obtain a light-yellow solid THPS@CS complex.

**Figure 2:**
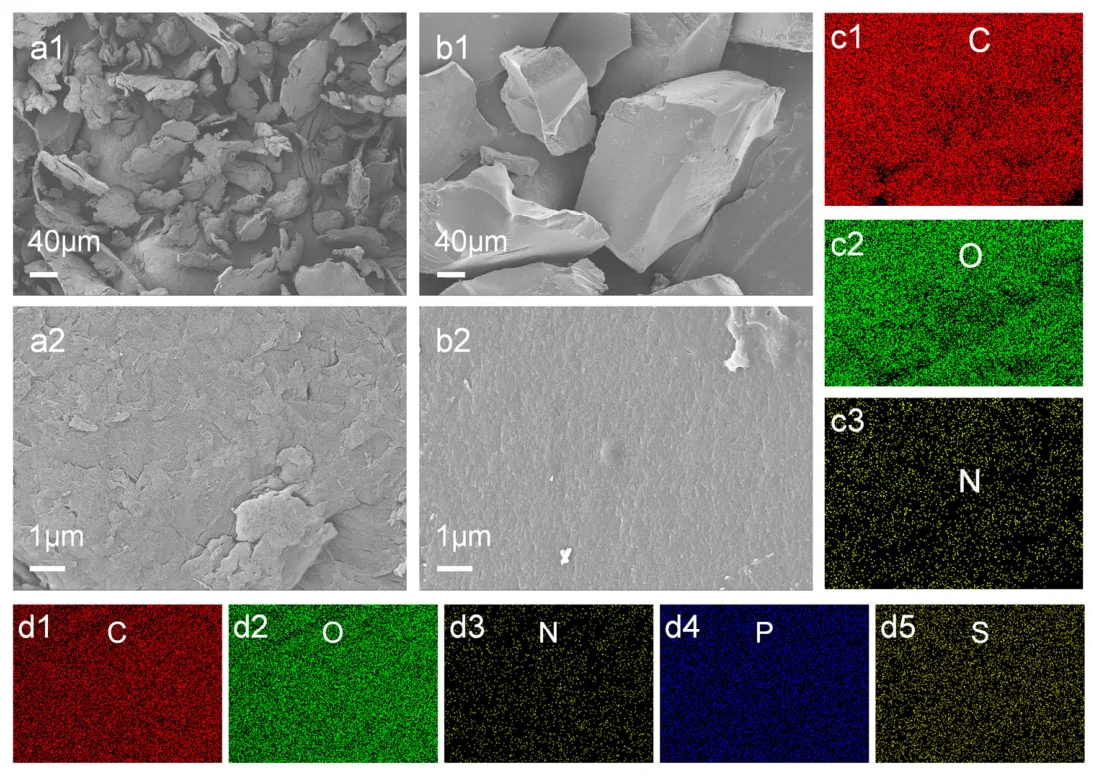
(**a1**) Low- and (**a2**) high-magnification FE-SEM images and (**c1**–**c3**) EDX elemental mapping images (C, O, N) of CS. (**b1**) Low- and (**b2**) high-magnification FE-SEM images and (**d1**–**d5**) EDX elemental mapping images (C, O, N, P, S) of THPS@CS.

Figure 2 displays FE-SEM images and corresponding EDX elemental mapping of CS and THPS@CS. CS consists of fragmented flakes and blocks with rough surfaces and abundant pores. In contrast, THPS@CS is transformed into regularly aggregated bulks featuring smoother and denser surfaces. This microstructural evolution essentially originates from the altered packing mode of CS chains after ionic complexation with THPS. EDX results reveal that only C, O, and N elements are detected in CS (see Figure 2(c1–c3)). In addition to C, O, and N elements, additional P and S elements originating from THPS are uniformly dispersed throughout THPS@CS (see Figure 2(d1–d5)). These observations verify the successful fabrication of THPS@CS complex, and the homogeneous dispersion of THPS favors the ultimate performance of resultant composites adhesive.

**Figure 3:**
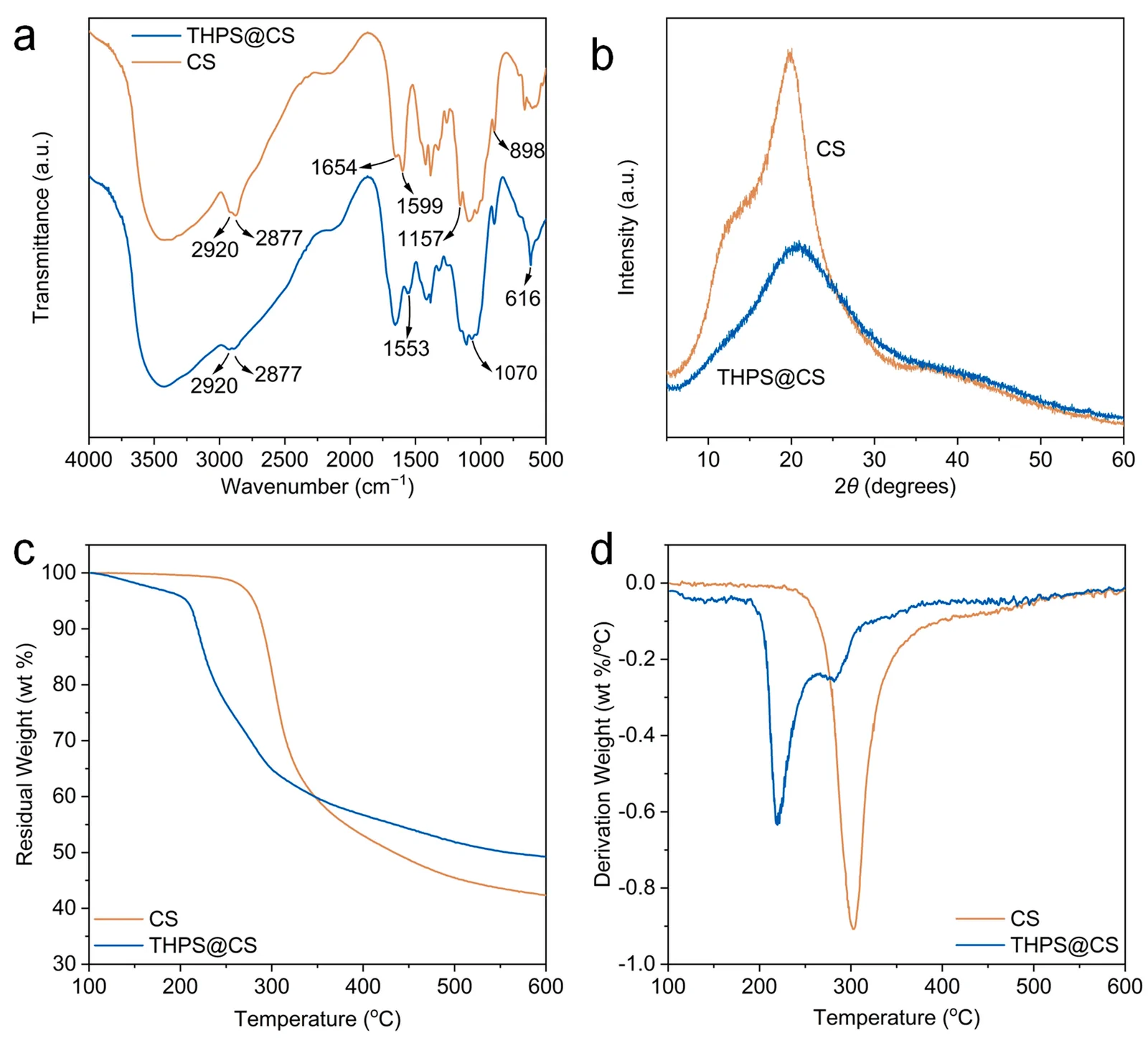
(**a**) FT−IR spectra, (**b**) XRD patterns, (**c**) TGA and (**d**) DTG curves under nitrogen condition of CS and THPS@CS.

Figure 3a shows the FT-IR spectra of CS and THPS@CS. Characteristic absorption peaks of CS appear at 2920 and 2877 cm^−1^ (C–H stretching of CH and CH_2_), 1654 cm^−1^ (amide I, C=O stretching of –CONH–), 1599 cm^−1^ (amide II, coupled N–H bending of –NH_2_ and C–N stretching), and 1157 and 898 cm^−1^ assigned to typical vibrations of the saccharide structure [18]. Compared with CS, the amide II peak of THPS@CS shifts to 1553 cm^−1^, providing evidence for ionic complexation [21]. Upon dissolution of CS in acetic acid, its –NH_2_ groups are protonated to form positively charged –NH_3_^+^. THPS, as a quaternary phosphonium salt, dissociates in aqueous solution into phosphonium cations [P(CH_2_OH)_4_]^+^ and sulfate counterions SO_4_^2−^. It is proposed that electrostatic attraction occurs between –NH_3_^+^ and SO_4_^2−^, which constructs the non-covalent ionic network of the as-formed polyelectrolyte hydrogel. Through such ionic interaction, THPS-derived phosphonium species are incorporated into the matrix. The distinct N–H microenvironment of –NH_3_^+^, relative to that of –NH_2_, explains the observed shift in the amide II peak. Furthermore, THPS@CS shows a new absorption peak at 616 cm^−1^, assigned to the characteristic vibration of SO_4_^2−^ from THPS, and another peak at 1070 cm^−1^, attributed to C–O stretching of the –CH_2_OH groups in THPS [22]. The reduced intensities at 2920 and 2877 cm^−1^ further suggest that intermolecular ionic complexation restricts the vibrational freedom of the alkyl chains in CS.

Figure 3b shows the XRD patterns of CS and THPS@CS. CS exhibits two characteristic diffraction peaks around 12° and 20°, arising from crystalline domains formed by ordered packing of its molecular chains via intermolecular hydrogen bonds [23]. Compared with CS, THPS@CS shows weakened and broadened diffraction peaks, indicating a remarkable decline in crystallinity [18]. This change is consistent with the occurrence of ionic complexation between CS and THPS, which disrupts the intrinsic hydrogen-bond network of CS, destroys the ordered arrangement of molecular chains, and reduces the fraction of crystalline regions.

Figure 3c,d present thermal degradation curves of CS and THPS@CS under a nitrogen atmosphere, and the detailed data are summarized in Table S2. For CS, the temperature at 5 wt% weight loss (*T*_5%_) is 280 °C, the temperature at maximum weight loss (*T*_max_) is 302 °C, the maximum mass loss rate (MLR_max_) reaches 0.91 wt%/°C, and the char residue at 600 °C is 42.4 wt%. In contrast, the *T*_5%_ of THPS@CS decreases to 207 °C, indicating earlier initial decomposition triggered by the incorporation of THPS. Its *T*_max_ drops to 220 °C, 82 °C lower than that of CS, while the MLR_max_ decreases to 0.64 wt%/°C. Meanwhile, the char yield of THPS@CS at 600 °C increases to 49.2 wt%, representing an increment of 6.8 wt% compared to CS. As the temperature increases to 350 °C, CS exhibits a markedly faster mass loss than THPS@CS. The DTG peak of CS is sharp and intense, whereas THPS@CS shows a weakened peak with a reduced MLR_max_. The sulfur- and phosphorus-containing THPS decomposes in advance upon heating, forming phosphorus-containing derivatives that may catalyze the decomposition of CS [17]. Although such catalytic effect reduces the initial decomposition temperature, it effectively suppresses the mass loss rate, facilitates char accumulation, and consequently improves the high-temperature thermal stability of the complex.

### 2.2. Characterization of SPI/WE/THPS@CS/MMT Composite Adhesive

According to the requirements for interior plywood adhesives specified in GB/T 9846–2015 (Plywood for General Use), dry and wet shear strengths are measured to determine the optimal WE loading in a series of SPI/WE composite adhesives, and the corresponding results are plotted in Figures S1 and S2. SPI presents dry and wet shear strengths of 1.18 MPa and 0.49 MPa, respectively. Both dry and wet shear strengths increase continuously as the WE dosage rises from 5 wt% to 40 wt%. For SPI/10WE, the dry shear strength reaches 1.25 MPa and the wet shear strength is up to 0.71 MPa, which exactly meets the threshold value required by GB/T 9846–2015. Notably, the optimization criterion in this work is not solely based on shear-strength performance. Although SPI/40WE exhibits the highest shear strength among these formulations, higher WE content brings practical concerns. First, commercial WE is substantially more expensive than SPI, and 40 wt% WE would increase raw-material cost and weaken the cost advantage of biomass-derived adhesives. Second, higher WE loading increases the fraction of combustible epoxy-related moieties within the adhesive matrix, which is unfavorable for developing high-performance flame-retardant adhesives. Moreover, although SPI/10WE meets the minimum standard requirement, it offers only a narrow strength safety margin for subsequent filler modification. As demonstrated by the later experimental results in Section 2.4, the incorporation of THPS@CS indeed induces a certain decrease in bonding strength. Taking bonding performance and available strength safety margin as the primary experimental evidence, combined with the above-mentioned practical constraints, 20 wt% WE is finally selected as the basic matrix for subsequent flame-retardant modification.

**Figure 4:**
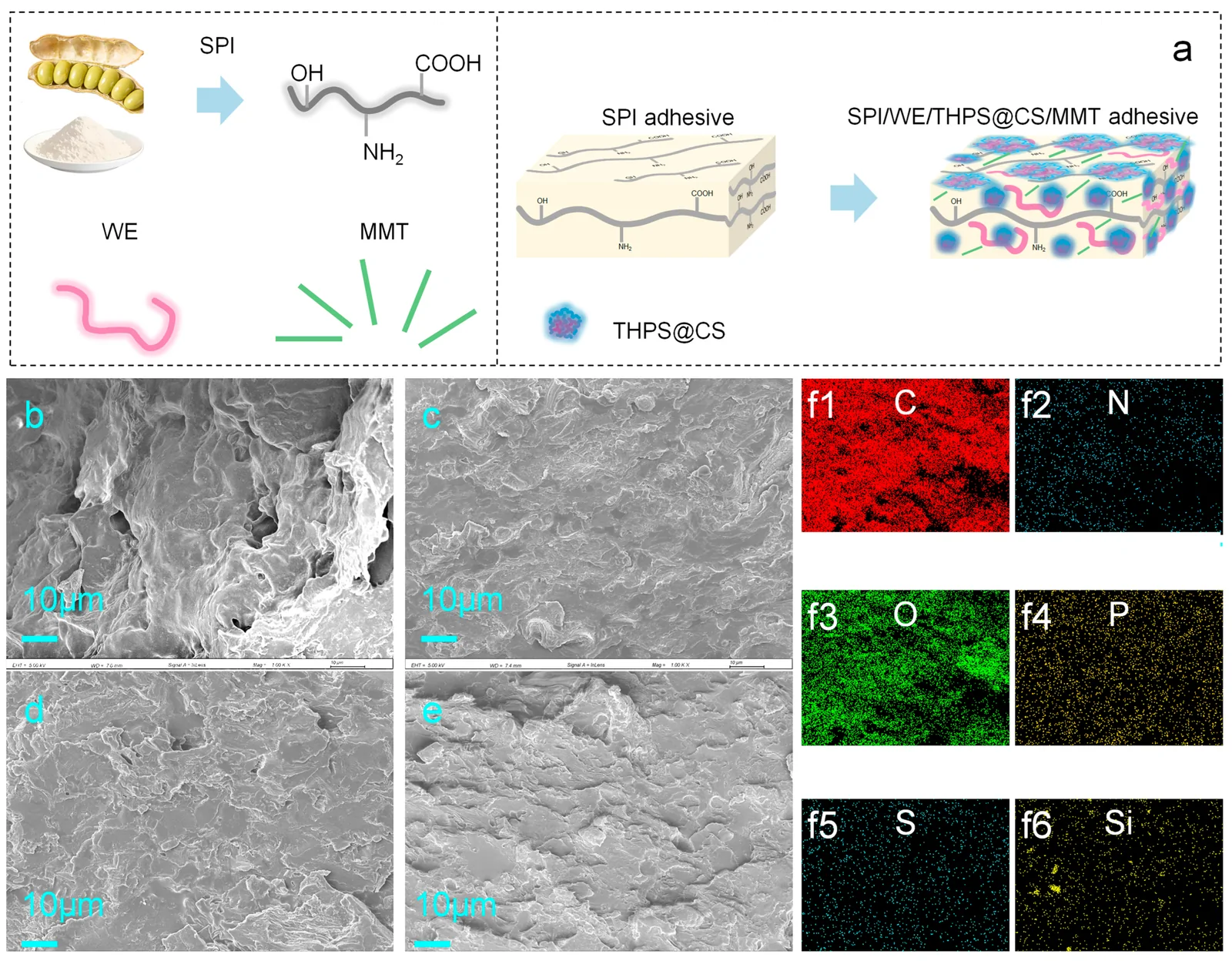
(**a**) Preparation illustration of SPI/WE/THPS@CS/MMT composite adhesives. FE-SEM images of (**b**) SPI, (**c**) SPI/20WE, (**d**) SPI/20WE/THPS@CS and (**e**) SPI/20WE/THPS@CS/MMT. (**f1**–**f6**) EDX elemental mapping images (C, N, O, P, S, Si) of SPI/20WE/THPS@CS/MMT.

Figure 4b–e display FE-SEM images of SPI, SPI/20WE, SPI/20WE/THPS@CS, and SPI/20WE/THPS@CS/MMT, together with EDX elemental mapping of SPI/20WE/THPS@CS/MMT. Many irregular voids are observed throughout SPI, indicative of its poor intrinsic cohesion [24]. Incorporation of WE reduces void content and improves structural continuity and compactness of SPI/20WE, demonstrating enhanced cohesive performance. The SPI/20WE/THPS@CS exhibits a homogeneous and compact morphology, implying favorable dispersion and interfacial compatibility of THPS@CS within the matrix. After further introducing MMT, SPI/20WE/THPS@CS/MMT forms a uniformly laminated stacking structure with improved structural integrity, which verifies the excellent dispersion of MMT. Moreover, EDX results reveal that P and S originating from THPS@CS as well as Si derived from MMT are homogeneously distributed across the SPI/20WE/THPS@CS/MMT (see Figure 4(f1–f6)). These results suggest that MMT nanolayers are well dispersed within the adhesive matrix, likely in a partially intercalated or exfoliated state rather than as large aggregates. This fine dispersion is essential for MMT to function as an effective physical barrier and to interact synergistically with the THPS@CS.

**Figure 5:**
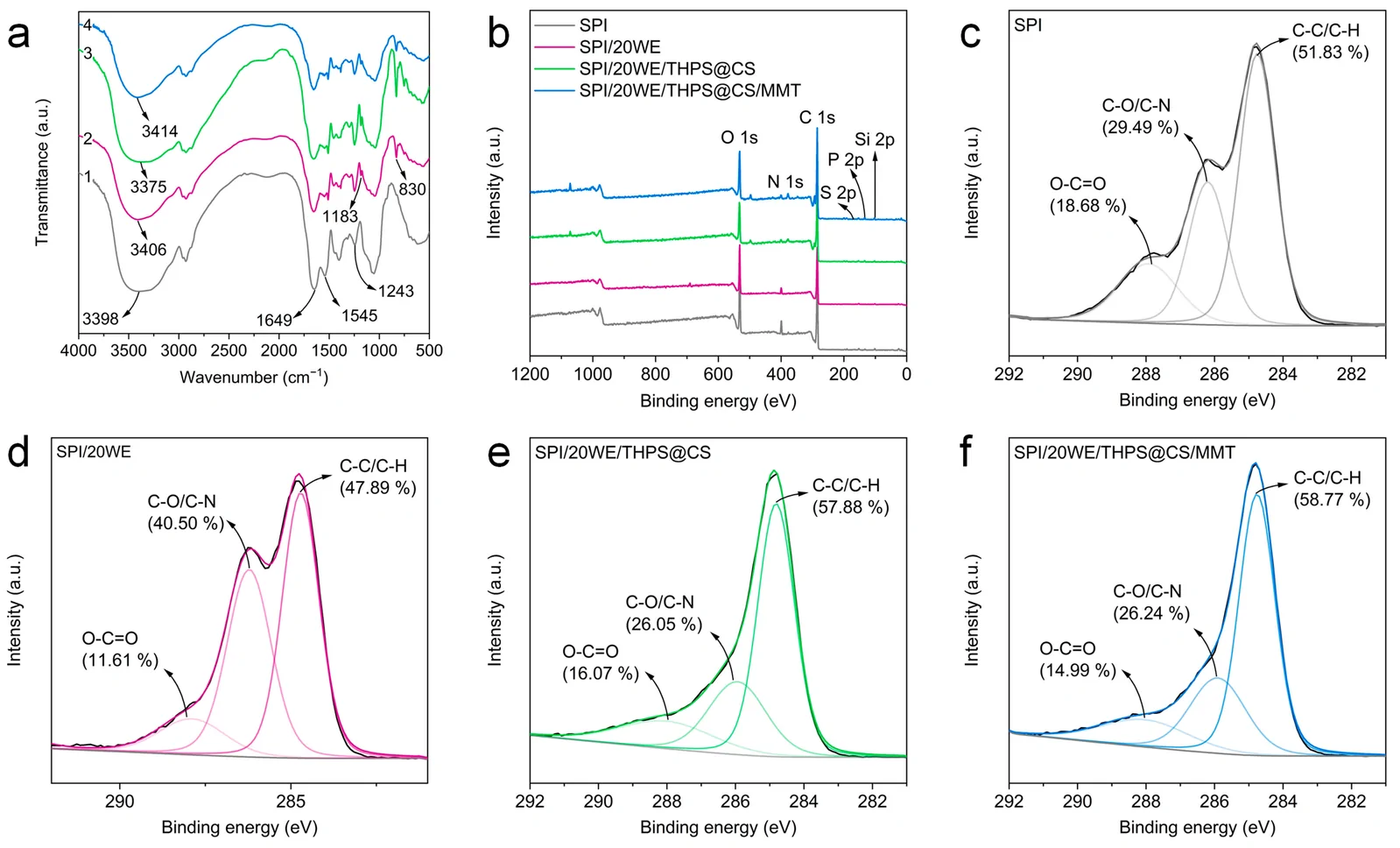
(**a**) FT-IR spectra of (1) SPI, (2) SPI/20WE, (3) SPI/20WE/THPS@CS, and (4) SPI/20WE/THPS@CS/MMT. (**b**) XPS spectra of SPI, SPI/20WE, SPI/20WE/THPS@CS, and SPI/20WE/THPS@CS/MMT. C 1s XPS spectra of (**c**) SPI, (**d**) SPI/20WE, (**e**) SPI/20WE/THPS@CS, and (**f**) SPI/20WE/THPS@CS/MMT.

Figure 5a shows the FT-IR spectra of SPI, SPI/20WE, SPI/20WE/THPS@CS, and SPI/20WE/THPS@CS/MMT. SPI exhibits characteristic absorption bands at 3398 cm^−1^ (overlapped stretching vibrations of O–H and N–H), 1649 cm^−1^ (amide I, C=O stretching), 1545 cm^−1^ (amide II, N–H bending) and 1243 cm^−1^ (amide III, coupled vibrations of N–H bending and C–N stretching) [6]. Compared with SPI, the overlapping O–H/N–H stretching band of SPI/20WE shifts to 3406 cm^−1^. This shift is attributed to intermolecular hydrogen bonding between the O–H/N–H groups of SPI and the –OH and C–O–C groups of ring-opened WE [25]. Meanwhile, the amide II peak splits into two separate bands, which is ascribed to ring-opening addition reactions between epoxy groups of WE and N–H groups of SPI, altering the chemical environment of N–H. Two new absorption peaks emerge near 1183 cm^−1^ and 830 cm^−1^, assigned to C–O–C stretching from ring-opened epoxy groups and para-substituted benzene ring vibrations of bisphenol-A-based WE, respectively [26]. For SPI/20WE/THPS@CS, the O–H/N–H stretching peak broadens and shifts to 3375 cm^−1^. This change arises from hydrogen-bond networks constructed via interactions between –CH_2_OH, SO_4_^2−^, and –NH_2_ groups of THPS@CS and polar groups on the SPI/20WE matrix [25]. The O–H/N–H band of SPI/20WE/THPS@CS/MMT further broadens and shifts to 3414 cm^−1^ owing to additional hydrogen bonding between surface hydroxyl groups on MMT nanosheets and O–H/N–H groups from SPI, WE, and THPS@CS [27].

Figure 5b and Table S3 show the elemental compositions and high-resolution C 1s XPS spectra of SPI, SPI/20WE, SPI/20WE/THPS@CS, and SPI/20WE/THPS@CS/MMT. For SPI, the percentages of C, N, and O are 72.2%, 5.0%, and 22.8%, respectively. After blending with WE, SPI/20WE features an elevated C content alongside reduced N and O fractions, which is attributed to the dilution effect of WE. In SPI/20WE/THPS@CS, the C content increases to 83.0% with further decreased N and O contents. Meanwhile, newly emerged P and S signals confirm the successful incorporation of THPS, and the abundant carbon chains of CS contribute to the increased C proportion. The detection of Si in SPI/20WE/THPS@CS/MMT directly verifies the presence of MMT.

As shown in Figure 5c–f, peak deconvolution of high-resolution C 1s spectra reveals three carbon configurations for SPI, namely C–C/C–H (51.83%), C–O/C–N (29.49%), and O–C=O (18.68%) [28]. In SPI/20WE, the fraction of C–C/C–H falls to 47.89%, while C–O/C–N rises to 40.50% and O–C=O decreases to 11.61%, originating from intermolecular interactions between SPI and WE that raise the proportion of heteroatom-bound carbon. SPI/20WE/THPS@CS shows a recovered C–C/C–H proportion of 57.88%, reduced C–O/C–N (26.05%) and increased O–C=O (16.07%). The CS in THPS@CS increase saturated hydrocarbon carbon, whereas the phosphonium groups and sulfate counterions from THPS interact with hydroxyl and amino groups of SPI and WE, thus modulating the chemical environment of carbonyl groups. For SPI/20WE/THPS@CS/MMT, the C–C/C–H content further increases with slight fluctuations in C–O/C–N and O–C=O contents, resulting from mild interactions between MMT and hydroxyl groups of the matrix that adjust carbon chemical states.

Figure S3 presents the insoluble fraction and moisture uptake values of SPI, SPI/20WE, SPI/20WE/THPS@CS, and SPI/20WE/THPS@CS/MMT. The insoluble fraction of SPI is 71.56%. In contrast, SPI/20WE achieves an increased value of 80.58%, because the epoxy groups of WE undergo ring-opening addition reactions with the amino and hydroxyl groups of SPI, constructing a robust chemically crosslinked network [11]. The insoluble fraction of SPI/20WE/THPS@CS further increases to 84.40%, which is ascribed to the densification effect of THPS@CS on the crosslinked network. The highest insoluble fraction of 85.13% is obtained for SPI/20WE/THPS@CS/MMT, owing to the physical barrier effect induced by laminated MMT nanosheets [19].

On the other hand, SPI exhibits a moisture uptake of 6.08%. In contrast, the value sharply rises to 22.15% for SPI/20WE, mostly due to the exposed hydrophilic groups of WE and continuous hydrophilic pathways inside the matrix that facilitate water adsorption and penetration. The moisture uptake of SPI/20WE/THPS@CS moderately decreases to 20.02%, because interfacial interactions between THPS@CS and the SPI/20WE matrix fill partial internal voids and cut off certain water diffusion channels. Benefiting from the impermeable barrier constructed by MMT, SPI/20WE/THPS@CS/MMT shows a further reduced moisture uptake of 11.62%, with suppressed water penetration and diffusion.

### 2.3. Flame Retardancy and Mechanism Analysis of Plywood

Two-layer plywood specimens are subjected to LOI to evaluate the flame retardancy of the above composite adhesives. As shown in Figure S4, raw wood has an LOI of 19.9%. Plywood bonded with SPI achieves an LOI of 24.4%, indicating improved flame retardancy. With SPI/20WE, the LOI increases to 25.3% because the epoxy groups of WE undergo ring-opening crosslinking with active groups of SPI, forming a compact network that suppresses combustible volatiles. The LOI further rises to 26.4% for SPI/20WE/CS, as CS dehydrates and carbonizes upon heating, generating a dense aromatic char layer that blocks heat and oxygen transfer [18]. For SPI/20WE/THPS@CS, the LOI reaches 27.6%, revealing a synergistic effect between THPS and CS. THPS provides phosphorus-containing moieties that catalyze crosslinking and char formation, while CS supports the char framework, enhancing structural integrity [17]. The highest LOI of 28% is obtained for SPI/20WE/THPS@CS/MMT, because layered MMT nanosheets promote regular stacking of the phosphorus-containing char, further improving barrier effect, and effectively delaying heat and oxygen transmission.

**Figure 6:**
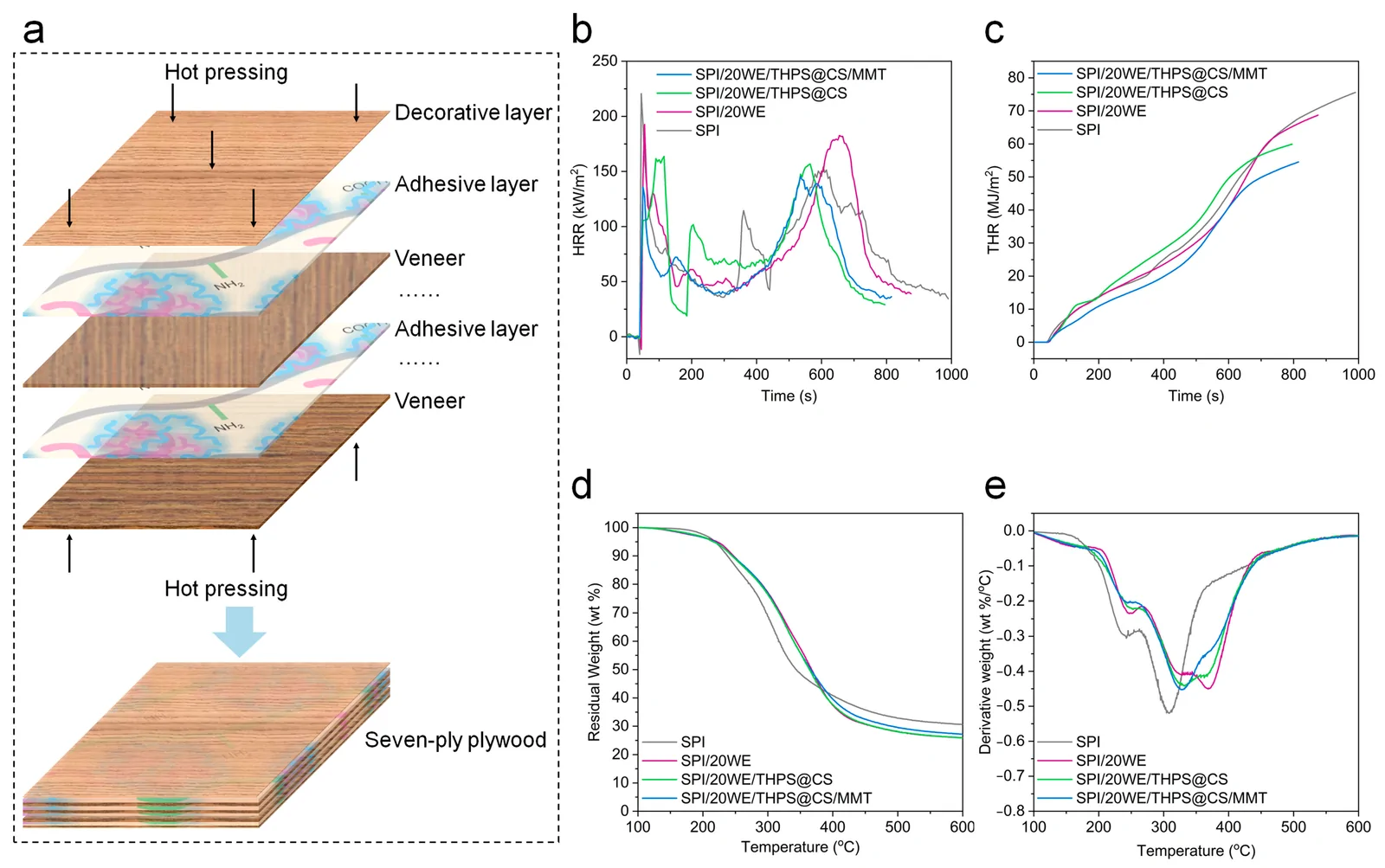
(**a**) Construction illustration of seven-ply plywood. (**b**) HRR and (**c**) THR curves of SPI, SPI/20WE, SPI/20WE/THPS@CS, and SPI/20WE/THPS@CS/MMT-bonded seven-ply plywood. (**d**) TGA and (**e**) DTG curves of SPI, SPI/20WE, SPI/20WE/THPS@CS, and SPI/20WE/THPS@CS/MMT under nitrogen condition.

Cone calorimeter measurements are also performed on seven-layer plywood, and the results are shown in Figure 6b,c. The heat release rate (HRR) curve of SPI-bonded plywood exhibits multiple peaks. After an initial peak of 221 kW/m^2^ at 45 s, the HRR drops sharply to 36 kW/m^2^, then rebounds to 114 kW/m^2^ at 360 s, drops again to 42 kW/m^2^, and finally rises gradually to 154 kW/m^2^ at 605 s. The total heat release (THR) is 75.6 MJ/m^2^. For plywood bonded with SPI/20WE, the first HRR peak is delayed to 55 s with a reduced peak value of 193 kW/m^2^, and a shoulder peak appears at 80 s (130.7 kW/m^2^). The second HRR peak disappears, whereas the third peak shifts to 660 s and increases to 183 kW/m^2^, accompanied by a decreased THR of 68.7 MJ/m^2^. These findings reveal that the incorporation of WE postpones combustion of plywood but aggravates heat release in the later stage. In contrast, SPI/20WE/THPS@CS-bonded plywood exhibits a further delayed first HRR peak at 115 s with a lower peak value of 163.6 kW/m^2^. Meanwhile, the second and third HRR peaks appear at 205 s and 565 s with reduced peak magnitudes of 101.7 kW/m^2^ and 156.8 kW/m^2^, respectively, and the corresponding THR drops to 59.9 MJ/m^2^. It demonstrates that THPS@CS significantly reduces THR, which can be explained by its prior decomposition during the second and third stages, forming a char layer that effectively hinders heat transfer. With the addition of MMT, the first HRR peak value of SPI/20WE/THPS@CS/MMT-bonded plywood decreases to 136.0 kW/m^2^, the second HRR peak disappears, the third HRR peak value decreases to 145.4 MJ/m^2^, and the THR is reduced to 54.5 MJ/m^2^, benefiting from the thermal barrier effect of layered MMT nanosheets.

To understand the difference in flame retardancy, the thermal degradation behaviors of SPI, SPI/20WE, SPI/20WE/THPS@CS and SPI/20WE/THPS@CS/MMT are examined using TGA. As shown in Figure 6d,e, SPI shows a *T*_5%_ of 222 °C, a *T*_max_ of 310 °C, and a char residue of 29.3 wt% at 600 °C. In comparison, SPI/20WE displays a reduced *T*_5%_ of 218 °C and a lower char yield of 25.1 wt% at 600 °C. Meanwhile, the *T*_max_ shifts to 320 °C accompanied by decreased MLR_max_. These results reveal that the incorporation of WE reduces the maximum mass loss rate owing to elevated crosslinking density [13], whereas the decreased char residue implies inferior char-forming capacity of WE relative to SPI. For SPI/20WE/THPS@CS, the *T*_5%_ further declines to 214 °C, which is ascribed to the earlier decomposition of phosphorus-containing groups [29]. The *T*_max_ shifts toward a higher temperature accompanied by a reduced MLR_max_, and the char residue at 600 °C rebounds to 25.7 wt%. These results confirm that THPS@CS suppresses the maximum mass loss rate and improves the char-forming efficiency. In contrast, SPI/20WE/THPS@CS/MMT achieves an increased *T*_5%_ of 219 °C and a higher char residue of 27.2 wt% at 600 °C, which originates from the improved compactness and thermal barrier performance of char layers induced by layered MMT.

**Figure 7:**
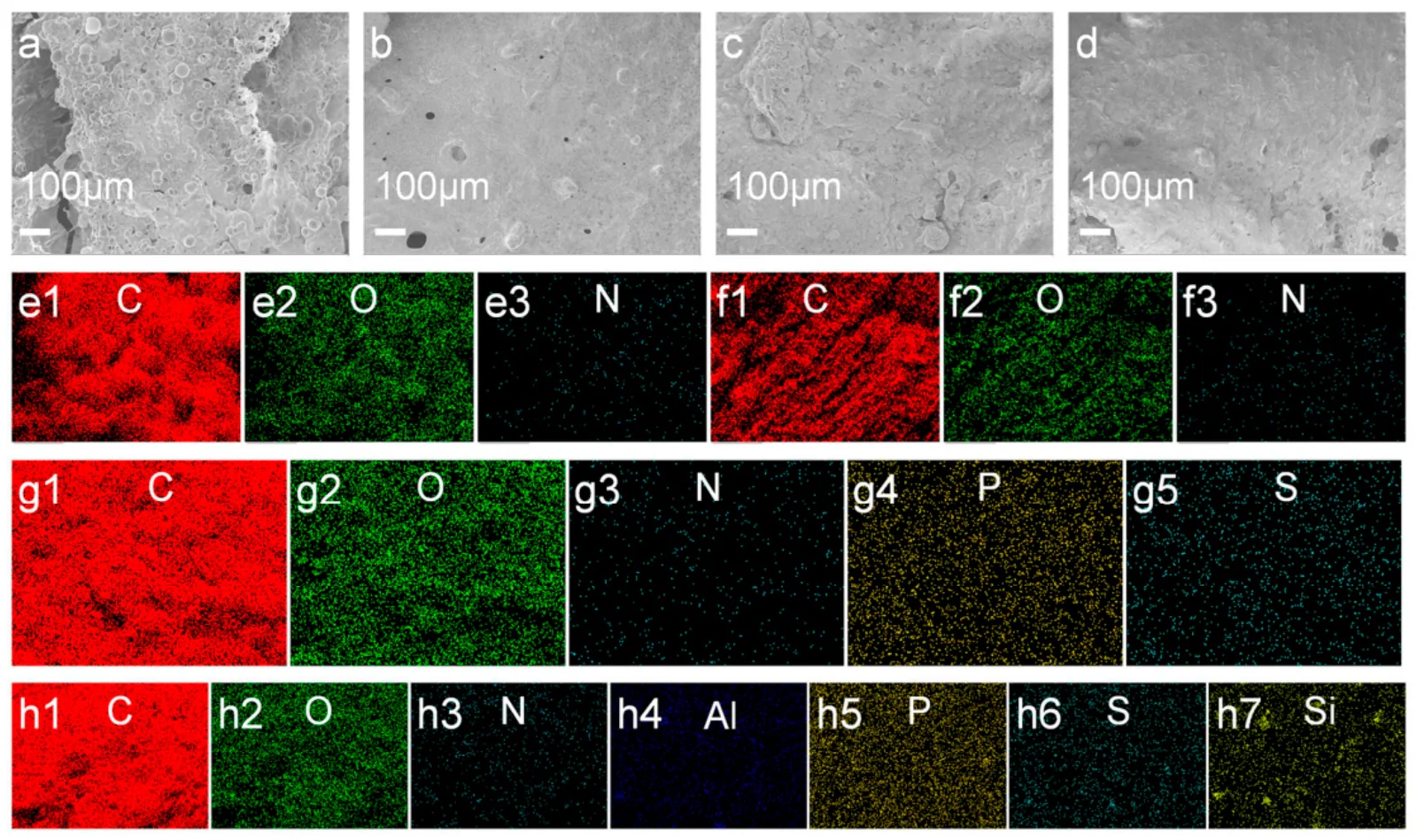
FE-SEM and EDX elemental mapping images of (**a**,**e1**–**e3**) SPI, (**b**,**f1**–**f3**) SPI/20WE, (**c**,**g1**–**g5**) SPI/20WE/THPS@CS, and (**d**,**h1**–**h7**) SPI/20WE/THPS@CS/MMT-bonded two-ply plywood after flame retardancy test.

Figure 7 presents FE-SEM images and EDX elemental mapping of char residues from burned plywood. The SPI-bonded plywood fails to form a continuous and compact char layer, and residual wood char can be seen through the cracks. In contrast, the char surface of plywood bonded with SPI/20WE becomes relatively smooth with only a few holes, indicating that WE helps make the char layer more continuous. The char derived from SPI/20WE/THPS@CS-bonded plywood shows reduced looseness and improved compactness. Moreover, the SPI/20WE/THPS@CS/MMT-bonded plywood produces an even smoother and tighter char layer. EDX elemental mapping reveals that only C, O, and N elements are detected in the char residue of SPI-bonded plywood. Similarly, C, O, and N remain the dominant elements for SPI/20WE-bonded plywood, indicating that WE does not introduce new elements into the char composition. Homogeneously dispersed P and S elements appear in the char of SPI/20WE/THPS@CS-bonded plywood, confirming that these elements migrate to the surface during combustion and participate in forming a condensed-phase protective char layer. In addition to P and S, uniformly distributed Si and Al signals are also observed in the char of SPI/20WE/THPS@CS/MMT-bonded plywood, which are attributed to MMT.

**Figure 8:**
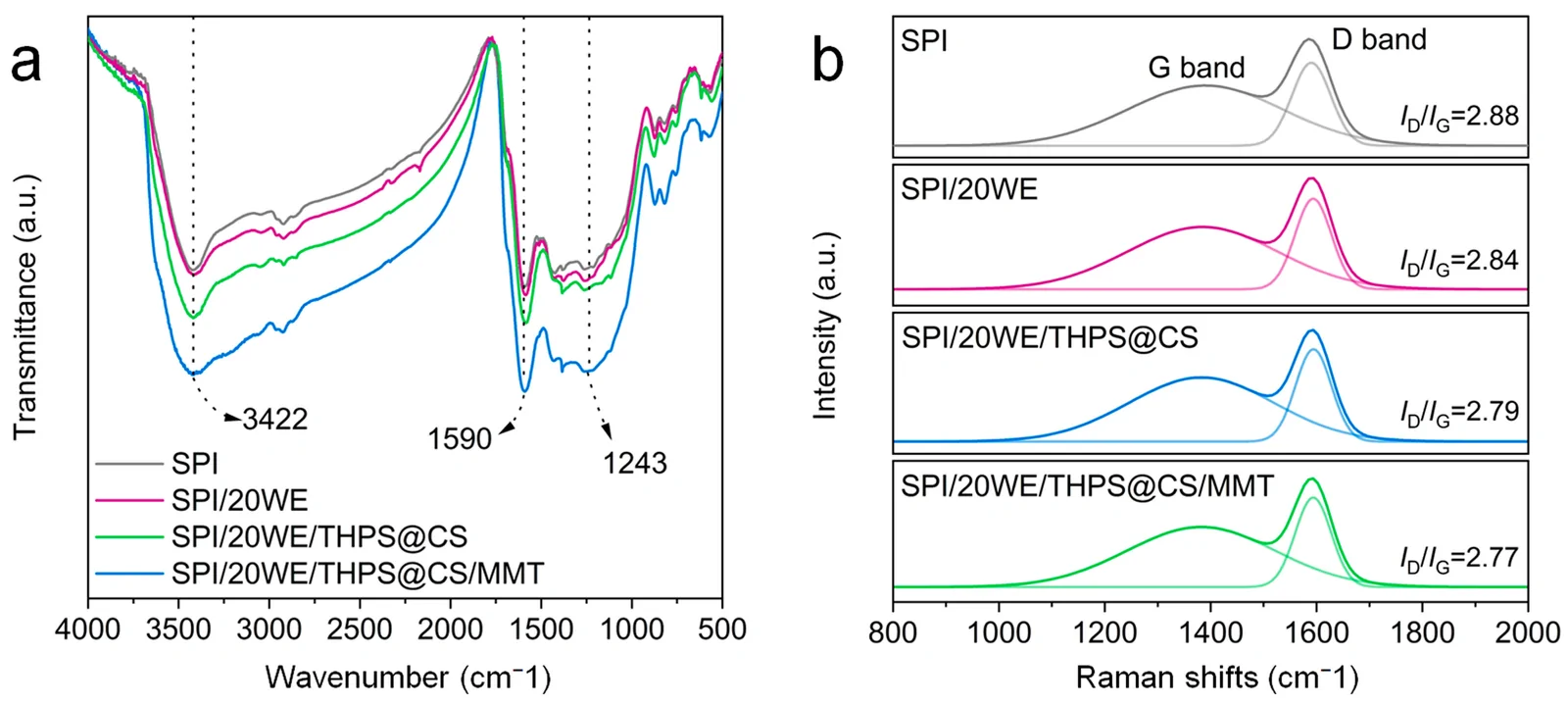
(**a**) FT-IR spectra and (**b**) Raman spectra of char residues from SPI-, SPI/20WE-, SPI/20WE/THPS@CS-, and SPI/20WE/THPS@CS/MMT-bonded plywood.

Figure 8a presents the FT-IR spectra of char residues from plywood after combustion. For the SPI-bonded plywood, most characteristic peaks assigned to protein structures nearly disappear, and a new peak emerges near 1590 cm^−1^ (C=C stretching of benzene rings), indicating severe decomposition and carbonization [30]. For the char of plywood bonded with SPI/20WE, the characteristic peaks originating from both protein and WE are greatly weakened, revealing that WE scarcely suppresses decomposition. In contrast, the char from SPI/20WE/THPS@CS-bonded plywood shows strengthened absorption peaks around 3422 (overlapped stretching vibrations of O–H and N–H), 1243 (amide III, coupled vibrations of N–H bending and C–N stretching) and 1590 cm^−1^, which demonstrates that THPS@CS suppresses decomposition and facilitates carbonization. These peak intensities further increase for the char of SPI/20WE/THPS@CS/MMT-bonded plywood, where more protein structures are retained.

Figure 8b shows the Raman spectra of char residues from burned plywood. All samples exhibit two characteristic peaks at 1350 cm^−1^ (D band) and 1580 cm^−1^ (G band). The D band corresponds to amorphous carbon, while the G band is associated with crystalline graphite carbon; the intensity ratio *I*_D_/*I*_G_ is widely used to evaluate the graphitization degree of residual char [31]. The *I*_D_/*I*_G_ values of char from plywood bonded with SPI and SPI/20WE are 2.88 and 2.84, respectively. After the addition of THPS@CS, the *I*_D_/*I*_G_ of char from SPI/20WE/THPS@CS-bonded plywood drops to 2.79, mainly because the phosphorus from THPS catalyze formation of carbon layers, and the carbon skeleton of CS supports the formation of graphitic carbon. The value further decreases to 2.77 for the char from SPI/20WE/THPS@CS/MMT-bonded plywood, as the layered MMT acts as a template to promote regular stacking of carbon and improves char graphitization.

**Figure 9:**
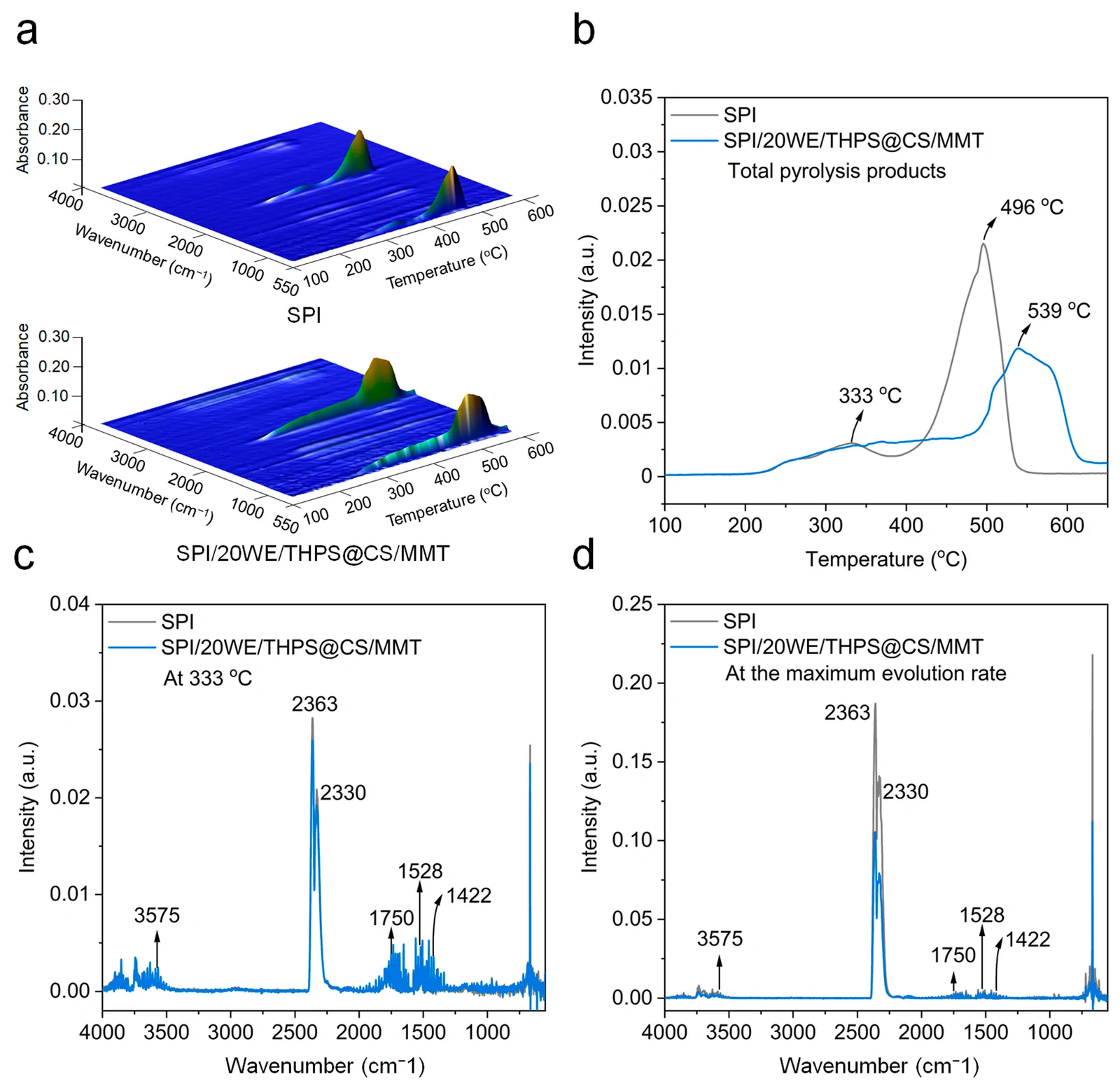
(**a**) 3D FT-IR spectra, (**b**) total pyrolysis products and FT-IR spectra obtained at (**c**) 333 °C and the (**d**) maximum evolution rates of SPI and SPI/20WE/THPS@CS/MMT under air atmosphere.

To identify the gaseous pyrolysis products released during oxidative degradation under combustion-like conditions, TG-FTIR is carried out under an air atmosphere for SPI and SPI/20WE/THPS@CS/MMT, and the results are displayed in Figure 9. According to the 3D FT-IR spectra (Figure 9a) and total pyrolysis products (Figure 9b), SPI releases gases in two distinct stages (around 333 °C and 496 °C). In contrast, SPI/20WE/THPS@CS/MMT shows a narrower release window with a single peak at 539 °C and lower intensity than the maximum peak of SPI, indicating that THPS@CS/MMT effectively delays the high-temperature decomposition of SPI. At 333 °C (Figure 9c), SPI/20WE/THPS@CS/MMT exhibits slightly higher peak intensities than SPI at 1750 cm^−1^ (C=O stretching), 1528 cm^−1^ (N–H bending) and 1422 cm^−1^ (C–H bending), implying earlier protein decomposition. At the temperature of maximum volatile release (Figure 9d), SPI/20WE/THPS@CS/MMT presents obviously weaker bands at 3575 cm^−1^ (O–H or N–H stretching), 2363 cm^−1^, and 2330 cm^−1^ (asymmetric stretching of CO_2_), as well as 1750 cm^−1^, 1528 cm^−1^, and 1422 cm^−1^ compared with SPI [32]. These results are mainly attributed to the combined catalytic charring and gas-dilution effects of THPS@CS/MMT, which suppress combustible volatile emission and convert most degradation products into stable char, thereby improving the thermal stability and flame retardancy of the composite adhesive.

**Figure 10:**
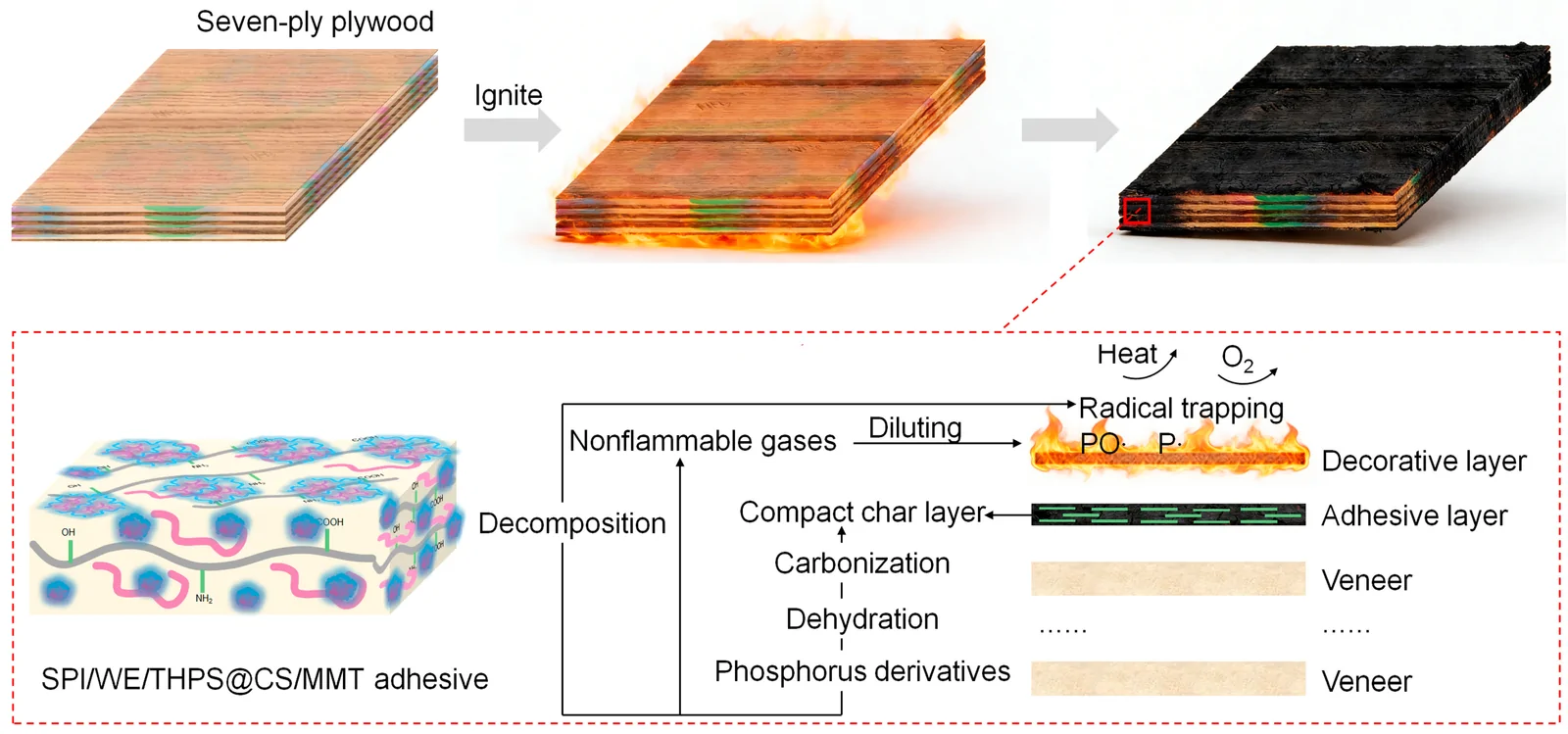
Flame retardant mechanism of SPI/20WE/THPS@CS/MMT.

Based on the above results and discussion, the flame retardancy of SPI/20WE/THPS@CS/MMT primarily originates from the condensed-phase catalytic charring effect of THPS@CS and barrier effect of MMT (see Figure 10). The phosphorus-containing derivatives decomposed from THPS@CS catalyze the charring of SPI, CS, and wood substrates, forming dense and compact char layers that effectively block heat and oxygen transfer [14]. MMT further complements the flame-retardant performance of THPS@CS via physical barrier effects. Layered MMT assembles into a brick-and-mortar structure, enhancing the high-temperature stability of char and further hindering heat and oxygen diffusion [33,34]. This barrier effect is consistent with the reduced moisture uptake and increased insoluble fraction (see Figure S3). FT-IR further confirms hydrogen bonding between surface hydroxyl groups on MMT and the adhesive matrix (see Figure 5a), ensuring good interfacial compatibility that facilitates integration of the rigid aluminosilicate framework into the char. Therefore, on the basis of current available evidence, the dominant contribution of MMT originates from its physical barrier function; whether MMT participates in catalytic aluminosilicate carbonization together with phosphorus species cannot be unambiguously verified in this study and remains an interesting direction for future investigation. In addition, according to previous literature reports [35,36], THPS@CS may exert potential gas-phase flame-retardant effects. It may release phosphorus radicals (e.g., PO•) to quench active H•/HO• radicals and terminate combustion chain reactions. Meanwhile, THPS@CS may generate sulfur-containing inert gases to dilute flammable volatiles and ambient oxygen, thereby inhibiting flame propagation.

### 2.4. Dry and Wet Shear Strength of SPI/WE/THPS@CS/MMT Composite Adhesive

**Figure 11:**
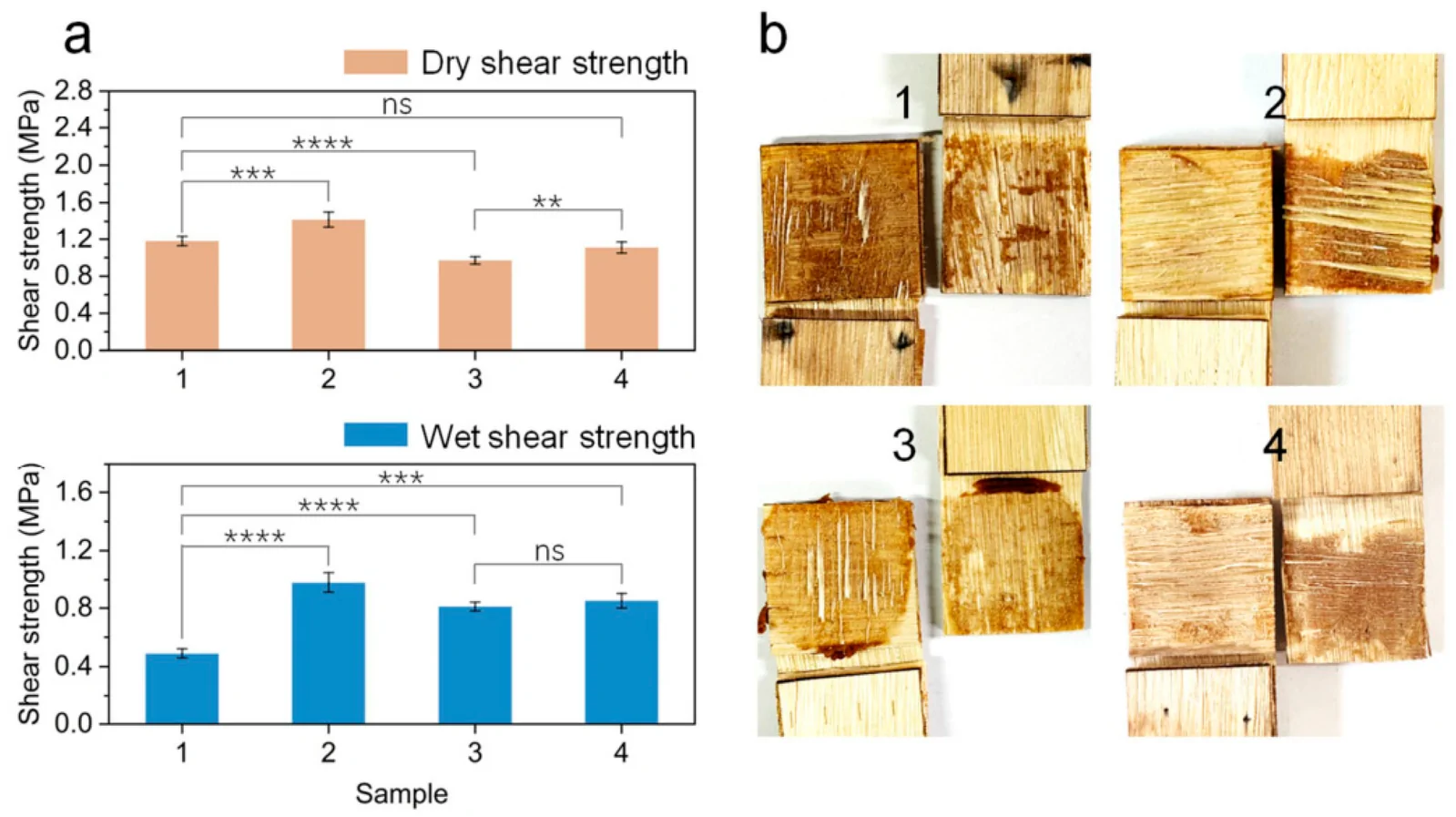
(**a**) Dry and wet shear strength and (**b**) wood failure rates in plywood specimens of (1) SPI, (2) SPI/20WE, (3) SPI/20WE/THPS@CS, and (4) SPI/20WE/THPS@CS/MMT. Error bars represent standard deviation (SD). ns: not significant; ** *p* < 0.01, ***p < 0.001, **** *p* < 0.0001.

As presented in Figure 11a, SPI exhibits a dry shear strength of 1.18 MPa and a wet shear strength of 0.49 MPa. SPI/20WE achieves improved dry and wet shear strengths of 1.42 MPa and 0.98 MPa, respectively. This improvement may be attributed to the crosslinking network formed by reactions between WE and active groups of SPI, which enhances cohesive strength and reduces water erosion at the adhesive–wood interface [10]. Figure 11b shows wood substrate failure, which provides a complementary qualitative indicator of bonding quality. Generally, a high wood substrate failure signifies that fracture occurs cohesively within the wood substrate rather than at the adhesive–wood interface [37], which is widely accepted as evidence of robust interfacial adhesion. It can be seen that the SPI/20WE shows extensive wood fiber rupture, indicating that the bonding strength exceeds the intrinsic cohesion of the wood. Notably, the improved wet shear strength is accompanied by an increase in moisture uptake (see Figure S3). This apparent contrast arises from the distinct mechanisms governing the two properties. Moisture uptake reflects the water absorption capacity of the adhesive matrix, and its increase in SPI/20WE is associated with the hydrophilic groups in WE. Wet shear strength, however, is determined by the cohesive strength of the adhesive and the integrity of the adhesive–wood interface, both of which are enhanced by the covalent crosslinking network [1]. Thus, the increased moisture uptake and improved wet strength are not mutually exclusive.

For SPI/20WE/THPS@CS, the dry and wet shear strengths drop to 0.97 MPa and 0.81 MPa, respectively. This decrease can be rationalized by the competitive crosslinking between WE and the multiple nucleophiles in the composite matrix. Both CS in THPS@CS and SPI possess –NH_2_ and –OH groups that can react with the epoxy groups of WE. Generally, –NH_2_ groups exhibit higher reactivity toward epoxy groups than –OH groups. The abundant –NH_2_ groups on CS may therefore preferentially consume a portion of the WE functionalities, reducing the effective crosslink density between WE and the SPI matrix. Additionally, the bulky polyelectrolyte complex structure of THPS@CS may impose steric hindrance, further impeding the formation of a uniform WE–SPI crosslink network. Consequently, the effective crosslink density of the adhesive matrix is diluted, leading to the observed reduction in shear strength. Nevertheless, the wet bonding strength still meets the minimum requirement (≥0.7 MPa) for interior plywood specified in GB/T 9846–2015.

Further incorporating MMT restores the dry shear strength from 0.97 MPa (SPI/20WE/THPS@CS) to 1.11 MPa (SPI/20WE/THPS@CS/MMT) (*p* < 0.01), confirming a statistically significant improvement in dry shear strength. This improvement can be attributed to the MMT nanosheets acting as physical reinforcing fillers dispersed in the adhesive matrix, effectively transferring and dissipating applied stress [19]. The surface –OH groups of MMT also form hydrogen bonds with the polar groups of SPI (as evidenced by the FT-IR in Figure 5a), thereby improving the interfacial adhesion and facilitating efficient stress transfer. In contrast, the wet shear strength shows only a slight increase from 0.81 MPa to 0.85 MPa after MMT incorporation, and this difference is not statistically significant (*p* > 0.05). The impermeable layered structure of MMT may create a tortuous diffusion path that hinders water penetration into the adhesive matrix [38], which is consistent with the lower moisture uptake measured for the MMT-containing adhesive (see Figure S3). However, this effect does not substantially improve the wet bonding strength. Therefore, the primary contribution of MMT to the adhesive performance lies in enhancing dry bonding strength, rather than achieving a notable improvement in wet-shear performance.

## 3. Conclusions

In conclusion, this work proposes a novel strategy that simultaneously endows plywood with high flame retardancy and favorable dry and wet shear strengths. Using this strategy, a novel polyelectrolyte-hydrogel-derived complex (THPS@CS) containing phosphorus, nitrogen, and sulfur is fabricated via ionic complexation and then incorporated into the SPI/20WE matrix together with MMT to obtain a composite adhesive (SPI/20WE/THPS@CS/MMT). The plywood bonded with this composite adhesive exhibits a maximum LOI of 28.0%, and cone calorimeter tests confirm its ability to effectively reduce heat release. The flame-retardant effect mainly originates from the phosphorus-containing derivatives generated by THPS decomposition, which catalyze the formation of a highly graphitized, dense char layer, while MMT acts as a physical barrier, reinforcing the stability and barrier performance of the char layer. In terms of bonding performance, the WE significantly improves the dry and wet shear strengths of SPI. The addition of THPS@CS causes slight fluctuations in strength, but subsequent incorporation of MMT restores and enhances the values, with both dry and wet shear strengths meeting the requirements of the standard for interior-use plywood specified in Plywood for General Use (GB/T 9846–2015). Therefore, this work provides new insights and a useful reference for the design and fabrication of bio-based plywood adhesives.

## 4. Materials and Methods

### 4.1. Materials

Bis[tetrakis(hydroxymethyl)phosphonium] sulfate solution (THPS, 75 wt% in water), chitosan (CS, deacetylation degree ≥ 95%), acetic acid (≥99%), montmorillonite (MMT, K-10), and sodium hydroxide (NaOH, ≥96%) were supplied by Aladdin Chemistry Co., Ltd. (Shanghai, China). Soybean protein isolate (SPI, protein content: ≥90 wt %, moisture content: ≤10 wt %) was bought from Anyang Tianxiangrui Food Technology Co., Ltd. (Anyang, China). A commercial waterborne epoxy resin (WE, F0704, 55 ± 2 wt %) and its curing agent (F0705, 44 ± 2 wt %) were bought from Shenzhen Jitian Chemical Co., Ltd. (Shenzhen, China). Poplar veneers (400 mm × 400 mm× 1.7 mm) and decorative veneers (400 mm × 400 mm × 0.4 mm) with a moisture content of approximately 20 wt% were bought from Jiangsu Guanyuan Home Co., Ltd. (Suqian, China). Deionized water with a resistance of 18 MΩ was utilized throughout the entire experimental procedure.

### 4.2. Preparation of THPS@CS Polyelectrolyte Complex

As shown in Figure 1, the 75 wt% THPS solution was diluted to 1 wt% using deionized water at room temperature. Dried CS powder was dissolved in a 1 wt% acetic acid solution under continuous magnetic stirring to prepare a 2 wt% CS solution. Then, 150 mL of 2 wt% CS solution (containing 3.0 g of CS) was added dropwise to 100 mL of 1 wt% THPS solution (containing 1.0 g of THPS) under constant magnetic stirring to form a polyelectrolyte hydrogel, corresponding to a THPS:CS mass ratio of 1:3. The as-obtained hydrogel was subsequently vacuum-dried and ground to produce THPS@CS powder with a yield of 96%.

### 4.3. Preparation of SPI/WE/THPS@CS/MMT Composite Adhesive

Firstly, the SPI powder was added to deionized water and stirred mechanically at 50 °C for 1 h. Then, a NaOH solution was added dropwise to adjust the pH to 10.0 and regulated the solid content of SPI to 30 wt%. The mixture was then stirred mechanically for a further hour to obtain a homogeneous SPI adhesive. Next, the WE dispersion and its curing agent (solid mass ratio of WE to curing agent = 2:1) were added to the SPI adhesive. The mixture was stirred mechanically until a uniform and stable system formed, yielding SPI/WE composite adhesives. The mass fractions of WE in the SPI/WE composites were set to 0 wt%, 5 wt%, 10 wt%, 20 wt%, and 40 wt%, and the corresponding samples were denoted as SPI, SPI/5WE, SPI/10WE, SPI/20WE, and SPI/40WE, respectively (Note: The WE content throughout this work was defined as the mass ratio of pure WE solid resin to the solid mass of SPI. The corresponding commercial WE dispersion and its curing agent were added based on their solid contents to achieve the desired solid loading. The curing agent was added at a fixed solid mass ratio of WE–curing agent = 2:1 and was not included in the WE dosage calculation).

Secondly, according to the shear strength presented in Figures S1 and S2, SPI/20WE composite adhesive was selected as the matrix. THPS, CS, THPS@CS, and ultrasonically dispersed MMT suspension and THPS@CS were separately incorporated into the SPI/20WE matrix, followed by mechanical stirring to form homogeneous and stable systems. Consequently, a series of composite adhesives including SPI/20WE/THPS, SPI/20WE/CS, SPI/20WE/THPS@CS, and SPI/20WE/THPS@CS/MMT were fabricated. The corresponding preparation process is illustrated in Figure 4a, and the detailed formulations are listed in Table S1.

### 4.4. Preparation of Plywood

Three plywood configurations were employed for different characterization purposes: two-layer plywood for LOI measurements, three-layer plywood for dry and wet shear-strengths tests, and seven-layer plywood for cone calorimeter measurements. It should be emphasized that direct cross-comparison of absolute performance values across two-layer, three-layer and seven-layer plywood was not carried out in this work. All comparisons among different adhesive formulations were performed within specimens of the identical plywood configuration for each given test.

#### 4.4.1. Preparation of Two-Layer Plywood

Two-layer plywood was fabricated by applying the composite adhesive to both sides of the veneer (180 g/m^2^ per side). Following a 15 min ambient open assembly, two veneers were stacked with their grain directions aligned parallel to each other, clamped with dovetail clips and dried at 60 °C for 24 h. The specimen surfaces were left as-prepared without sanding, retaining the adhesive layer on both surfaces. The resulting plywood was conditioned at 25 °C and 60% RH for 24 h prior to testing. It should be noted that the two-layer plywood was prepared without hot-pressing. This was because direct contact between the hot-press platens and the adhesive-coated veneer surface would cause the adhesive layer to adhere to the platens and peel off from the veneer, resulting in an uneven and incomplete adhesive coating on the specimen surface.

#### 4.4.2. Preparation of Three-Layer Plywood

Three-layer plywood was fabricated by coating the core veneer with the composite adhesive (180 g/m^2^ per side). After 15 min of ambient open assembly, the coated core veneer was sandwiched between two uncoated veneers with grain directions alternating perpendicularly. The assembly was hot-pressed at 120 °C and 1.0–1.5 MPa for 15 min, cold-pressed for 30 min and dried at 60 °C for 24 h. The plywood was conditioned at 25 °C and 60% RH for 24 h before testing.

#### 4.4.3. Preparation of Seven-Layer Plywood

Seven-layer plywood was fabricated as depicted in Figure 6a. The composite adhesive was spread onto both sides of the veneers at 180 g/m^2^ per side. After 15 min at room temperature, five core veneers and two decorative veneers (as the top and bottom faces) were stacked in alternating perpendicular grain directions. The assembly was hot-pressed at 120 °C and 1.0–1.5 MPa for 15 min, cold-pressed for 30 min and dried at 60 °C for 24 h. The resulting plywood was conditioned at 25 °C and 60% RH for 24 h before testing.

### 4.5. Characterization

Fourier transform infrared (FT-IR) spectra were acquired using a Thermo−Nicolet iS50 spectrometer (16 scans, resolution 4 cm^−1^, Thermo−Nicolet, Madison, WI, USA). X-ray diffraction (XRD) patterns were collected on a Bruker D8 ADVANCE X-ray diffractometer (Cu Kα radiation, λ = 0.154 nm, Bruker, Billerica, MA, USA) over a 2θ range of 5–60°. Morphology and elemental composition were characterized using a ZEISS Gemini SEM360 field-emission scanning electron microscopy (FE−SEM) fitted with an energy-dispersive X-ray (EDX) analyzer (Carl Zeiss AG, Oberkochen, Baden-Württemberg, Germany). Thermogravimetric analysis (TGA) was performed under nitrogen atmosphere using a NETZSCH STA 2500 thermogravimetric analyzer (NETZSCH, Selb, Bavaria, Germany) to evaluate the intrinsic thermal stability. X-ray photoelectron spectroscopy (XPS) analysis was performed on a Thermo Scientific ESCALAB 250Xi X-ray spectrometer (Thermo Fisher Scientific, Waltham, MA, USA). To determine the insoluble fraction, measurements were performed in triplicate. The cured adhesive was first dried at 120 °C to constant weight (W_1_). It was then immersed in deionized water at 60 °C for 6 h and dried again at 120 °C to constant weight (W_2_). The insoluble fraction was calculated as (W_2_/W_1_) × 100%. To determine the moisture uptake, measurements were performed in triplicate. The cured adhesive was first dried at 120 °C to constant weight (W_1_). It was then placed in a humidity chamber at 50 °C and 100% RH until constant weight (W_3_). The moisture uptake was calculated as (W_3_ − W_1_)/W_1_ × 100%. Limiting oxygen index (LOI) values for the two-layer plywood were obtained using a Phoenix PX-01-005 oxygen index tester (Phoenix, Suzhou, Jiangsu, China) in compliance with ASTM D2863 [39] (Standard Test Method for Measuring the Minimum Oxygen Concentration to Support Candle-Like Combustion of Plastics). Although ASTM D2863 was originally developed for plastics, its principle of measuring the minimum oxygen concentration supporting sustained combustion was applied to the two-layer plywood, as LOI offers a reproducible measure of flammability. The two-layer plywood was selected because it allows direct exposure of both adhesive-coated surfaces to the flame and enables rapid comparative evaluation of different formulations. Each specimen (130 mm × 65 mm × 4 mm, with the 130 mm dimension oriented along the wood grain) was mounted vertically in the combustion column and ignited from the top with a propane flame. The oxygen/nitrogen ratio was adjusted to determine the minimum oxygen concentration sustaining combustion for 3 min or a burn length of 50 mm. The test was conducted with a minimum of five replicates per sample to ensure reproducibility. The flammability of seven-layer plywood (100 mm × 100 mm × 9 mm) was assessed using a Fire Testing Technology cone calorimetry (FTT Cone2+) under a heat flux of 50 kW/m^2^ according to ISO 5660-1 [40] (Reaction-to-fire tests—Heat release, smoke production and mass loss rate Part 1: Heat release rate (cone calorimeter method) and smoke production rate (dynamic measurement)). The seven-layer plywood was selected to better simulate the fire response of real commercial plywood products, as it more closely represents actual plywood in terms of thickness and heat-transfer behavior. Each specimen was wrapped in aluminum foil on the back and edges, leaving only the top surface exposed, and placed horizontally in the specimen holder with the exposed surface facing the conical heater. The test was conducted with a minimum of three replicates per sample to ensure reproducibility. Raman spectroscopy was performed at room temperature on a Horiba Jobin-Yvon LabRAM HR800 Raman microscope (Horiba Jobin-Yvon, Longjumeau, Essonne, France). The pyrolysis product was examined by a NETZSCH STA 2500 thermogravimetric analyzer (NETZSCH, Selb, Bavaria, Germany) coupled with a Thermo−Nicolet iS50 spectrometer (TGA−FTIR, Thermo−Nicolet, Madison, WI, USA) in an air atmosphere to simulate the oxidative degradation behavior during combustion. The dry and wet shear strengths of three-layer plywood (length × width: 100 mm × 25 mm, glue area: 25 mm × 25 mm) were evaluated according to Chinese national standard GB/T 17657-2013 [41] (Test methods of evaluating the properties of wood-based panels and surface decorated wood-based panels), which provides the test procedure for determining the shear strength. This standard technically aligns with the international standard ISO 12466-1 [42] (Plywood-Bonding quality-Part 1: Test methods). The three-layer plywood was selected to comply with the specimen geometry specified in GB/T 17657-2013 [41], which is the standard method for evaluating the bonding performance of plywood. Tests were performed at room temperature using a Lishi LD25.504 universal testing machine at a crosshead speed of 10 mm/min, with six replicate specimens per formulation. The measured values were then evaluated against the performance requirement specified in GB/T 9846-2015 (Plywood for general use), which sets a minimum shear strength of 0.7 MPa for interior-use (Class II) plywood. For the wet shear strength measurement, the three-layer plywood specimens were soaked in deionized water at 63 °C for 3 h and then allowed to cool naturally to 25 °C prior to testing.

### 4.6. Statistical Analysis

All data analyses were performed using Microsoft Excel 2019 (Microsoft, Redmond, WA, USA). Experimental data are uniformly expressed as the mean ± standard deviation (SD). One-way analysis of variance (ANOVA) coupled with Tukey’s post-hoc test was applied for statistical comparison among groups. Differences were regarded as significant when *p* < 0.05.

## Data Availability

The data that support the findings of this study are available from the corresponding authors upon reasonable request.

## Supplementary Materials

The following supporting information can be downloaded at https://www.mdpi.com/article/10.3390/gels12090834/s1, Figure S1: Dry shear strength values of (a) SPI, (b) SPI/5WE, (c) SPI/10WE, (d) SPI/20WE and (e) SPI/40WE. Error bars represent standard deviation (SD); Figure S2. Wet shear strength values of (a) SPI, (b) SPI/5WE, (c) SPI/10WE, (d) SPI/20WE and (e) SPI/40WE. Error bars represent standard deviation (SD); Figure S3. Insoluble fraction and moisture uptake values of (a) SPI, (b) SPI/20WE, (c) SPI/20WE/THPS@CS and (d) SPI/20WE/THPS@CS/MMT. Error bars represent standard deviation (SD); Figure S4. LOI values of (a) untreated wood, and the two-ply plywood (130 mm × 65 mm × 4 mm) bonded with (b) SPI, (c) SPI/20WE, (d) SPI/20WE/CS, (e) SPI/20WE/THPS@CS and (f) SPI/20WE/THPS@CS/MMT; Table S1: Adhesive formulations of SPI/WE/THPS@CS/MMT; Table S2: Thermal degradation behaviors of CS and THPS@CS under nitrogen condition; Table S3: Elemental compositions of SPI, SPI/20WE, SPI/20WE/THPS@CS and SPI/20WE/THPS@CS/MMT.

## References

[1] Oni O.V., Lawrence M.A., Zappi M.E., Chirdon W.M. (2023). A Review of Strategies to Enhance the Water Resistance of Green Wood Adhesives Produced from Sustainable Protein Sources. Sustainability.

[2] Schick L.W., Zuo S., Faga M., Pai A.R., Chitsomkhuan S., Jaworski A., Cracco D., Marson A., Manzardo A., Samec J.S.M. (2026). Flame-retardant wood adhesive prepared from pulp mill residues. Green Chem..

[3] Kamperidou V., Akritidou V., Barboutis I. (2026). Bonding Performance of Natural Protein-Based Adhesive Systems on European Beech Wood Under Ambient and Moderate Thermal Exposure. Forests.

[4] Gu W., Li F., Liu X., Gao Q., Gong S., Li J., Shi S.Q. (2020). Borate chemistry inspired by cell walls converts soy protein into high-strength, antibacterial, flame-retardant adhesive. Green Chem..

[5] Yang G., Huang X., Cai J., Zhang Q. (2021). Curing mechanism of triglycidylamine crosslinked soybean protein adhesive analyzed by Fourier transform infrared, second derivative infrared and two-dimensional correlation spectroscopy. Int. J. Adhes. Adhes..

[6] Zeng Y., Yang W., Xu P., Cai X., Dong W., Chen M., Du M., Liu T., Jan Lemstra P., Ma P. (2022). The bonding strength, water resistance and flame retardancy of soy protein-based adhesive by incorporating tailor-made core–shell nanohybrid compounds. Chem. Eng. J..

[7] Jiang K., Dong X., Chen Y., Fan D., Chu F. (2024). A Room-Temperature Curing Plant Protein-Based Adhesive with High Strength and Flame Retardancy for Heat-Free Adhesion. Adv. Funct. Mater..

[8] Wang Z., Chen Y., Chen S., Chu F., Zhang R., Wang Y., Fan D. (2019). Preparation and characterization of a soy protein based bio-adhesive crosslinked by waterborne epoxy resin and polyacrylamide. RSC Adv..

[9] Lu W., Zhang S., Wang L., Guo C., Wang X., Wang D., Zhao Z.-B., Yang K., Ma Y., Li W. (2022). Understanding the role of epoxy emulsifiers in water-borne epoxy coatings with the aggregation-induced emission approach. Prog. Org. Coat..

[10] Sun Y., Yan Q., Liang Z., Zhang S., Kang H. (2024). Synthesis of soy-protein adhesives with excellent cohesion and cold-pressing bonding strength through multiple noncovalent crosslinking network of waterborne epoxy resin. Constr. Build. Mater..

[11] Pang H., Zhao S., Wang Z., Zhang W., Zhang S., Li J. (2020). Development of soy protein-based adhesive with high water resistance and bonding strength by waterborne epoxy crosslinking strategy. Int. J. Adhes. Adhes..

[12] Solis-Pomar F., Díaz-Gómez A., Berrío M.E., Ramírez J., Jaramillo A.F., Fernández K., Rojas D., Melendrez M.F., Pérez-Tijerina E. (2021). A Dual Active-Passive Coating with Intumescent and Fire-Retardant Properties Based on High Molecular Weight Tannins. Coatings.

[13] Ruan L., Hu D., Pan Z., Zhang X., Yu K., Ma W. (2024). Novel eco-friendly bio-nano flame retardant via the electrostatic-driven hybridization of natural halloysite nanotubes and phytic acid for superior fire safety of epoxy composites. Compos. Commun..

[14] Huo S., Song P., Yu B., Ran S., Chevali V.S., Liu L., Fang Z., Wang H. (2021). Phosphorus-containing flame retardant epoxy thermosets: Recent advances and future perspectives. Prog. Polym. Sci..

[15] Li X., Wang Y., Chen Y., Du J., Chen Y., Yu H., Huo J., Tan X., Feng Y. (2025). A hyperbranched P/N/B/Si-containing multi-element flame retardant for high performance and fire safety epoxy thermosets. Polym. Degrad. Stab..

[16] Aleksandr K., Mikhail L., Aleksandr P. (2024). Self-Assembled Hydrogel Based on (Bio)polyelectrolyte Complex of Chitosan-Gelatin: Effect of Composition on Physicochemical Properties. Gels.

[17] Diao S., Liang M., Lu Y., Yang Y., Tang Q., Zhang G. (2025). A high-molecular-weight flame retardant derived from bis[tetrakis(hydroxymethyl)phosphonium] sulfate for cotton fabrics. Polym. Degrad. Stab..

[18] Zhang T., Yan H., Shen L., Fang Z., Zhang X., Wang J., Zhang B. (2014). Chitosan/Phytic Acid Polyelectrolyte Complex: A Green and Renewable Intumescent Flame Retardant System for Ethylene–Vinyl Acetate Copolymer. Ind. Eng. Chem. Res..

[19] Bian Y., Guo Q., Cao J., Li J. (2025). Soy Protein Adhesive Enhanced Through Supramolecular Interaction with Tannic Acid Modified Montmorillonite. Chem. Eur. J..

[20] (2015). Plywood for General Use.

[21] Sing C.E., Perry S.L. (2020). Recent progress in the science of complex coacervation. Soft Matter.

[22] Liang S., Cai W., Dang C., Peng X., Luo Z., Wei X. (2023). Synthesis of sodium alginate/phosphorus tetramethylmethyl sulfate biocomposite beads with exceptional adsorption rate for Cr(VI) removal. J. Environ. Chem. Eng..

[23] Pereira M.B.B., Franca D.B., Araujo R.C., Silva Filho E.C., Rigaud B., Fonseca M.G., Jaber M. (2020). Amino hydroxyapatite/chitosan hybrids reticulated with glutaraldehyde at different pH values and their use for diclofenac removal. Carbohydr. Polym..

[24] Zhao X., Liu T., Ou R., Hao X., Fan Q., Guo C., Sun L., Liu Z., Wang Q. (2022). Fully Biobased Soy Protein Adhesives with Integrated High-Strength, Waterproof, Mildew-Resistant, and Flame-Retardant Properties. ACS Sustain. Chem. Eng..

[25] Song P., Dai J., Chen G., Yu Y., Fang Z., Lei W., Fu S., Wang H., Chen Z.-G. (2018). Bioinspired Design of Strong, Tough, and Thermally Stable Polymeric Materials via Nanoconfinement. ACS Nano.

[26] Pradhan S., Mohanty S., Nayak S.K. (2017). Waterborne epoxy adhesive derived from epoxidized soybean oil and dextrin: Synthesis and characterization. Int. J. Polym. Anal. Charact..

[27] Tien Y.I., Wei K.H. (2001). Hydrogen bonding and mechanical properties in segmented montmorillonite/polyurethane nanocomposites of different hard segment ratios. Polymer.

[28] Pang H., Ma C., Shen Y., Sun Y., Li J., Zhang S., Cai L., Huang Z. (2021). Novel Bionic Soy Protein-Based Adhesive with Excellent Prepressing Adhesion, Flame Retardancy, and Mildew Resistance. ACS Appl. Mater. Interfaces.

[29] Huo S., Sai T., Ran S., Guo Z., Fang Z., Song P., Wang H. (2022). A hyperbranched P/N/B-containing oligomer as multifunctional flame retardant for epoxy resins. Compos. Part B Eng..

[30] Zhang T., Yan H., Peng M., Wang L., Ding H., Fang Z. (2013). Construction of flame retardant nanocoating on ramie fabric via layer-by-layer assembly of carbon nanotube and ammonium polyphosphate. Nanoscale.

[31] Dai J., Xu Y., Zhang T., Zhang X. (2026). Epoxidized Soybean Oil-Based Phosphorus-Containing Waterborne Polyurethane Coating for PET Rope With Enhanced Flame Retardancy and Mechanical Properties. J. Appl. Polym. Sci..

[32] Loría-Bastarrachea M.I., Herrera-Kao W., Cauich-Rodríguez J.V., Cervantes-Uc J.M., Vázquez-Torres H., Ávila-Ortega A. (2010). A TG/FTIR study on the thermal degradation of poly(vinyl pyrrolidone). J. Therm. Anal. Calorim..

[33] Ding F., Liu J., Zeng S., Xia Y., Wells K.M., Nieh M.-P., Sun L. (2017). Biomimetic nanocoatings with exceptional mechanical, barrier, and flame-retardant properties from large-scale one-step coassembly. Sci. Adv..

[34] Beduini A., Carosio F., Ferruti P., Ranucci E., Alongi J. (2024). Synergism between α-amino acid-derived polyamidoamines and sodium montmorillonite for enhancing the flame retardancy of cotton fabrics. Polym. Degrad. Stab..

[35] Zilke O., Ali W., Kamps L., Engels T., Schumacher S., Danielsiek D., Shabani V., Salma A., Plohl D., Wallmeier R. (2022). Water-Soluble Cyclophosphazenes as Durable Flame-Retardant Finishes for Nylon/Cotton Blend Fabrics. ACS Appl. Polym. Mater..

[36] Pellerin S., Samyn F., Duquesne S., Landry V. (2022). Preparation and Characterisation of UV-Curable Flame Retardant Wood Coating Containing a Phosphorus Acrylate Monomer. Coatings.

[37] Alade A.A., Naghizadeh Z., Wessels C.B. (2022). A new method for estimating wood failure percentage in adhesive-bonded shear specimens. Int. J. Adhes. Adhes..

[38] Garusinghe U.M., Varanasi S., Raghuwanshi V.S., Garnier G., Batchelor W. (2018). Nanocellulose-montmorillonite composites of low water vapour permeability. Colloids Surf. A Physicochem. Eng. Asp..

[39] (2019). Standard Test Method for Measuring the Minimum Oxygen Concentration to Support Candle-Like Combustion of Plastics (Oxygen Index).

[40] (2015). Reaction-to-Fire Tests—Heat Release, Smoke Production and Mass Loss Rate—Part 1: Heat Release Rate (Cone Calorimeter Method) and Smoke Production Rate (Dynamic Measurement).

[41] (2013). Test Methods of Evaluating the Properties of Wood-Based Panels and Surface Decorated Wood-Based Panels.

[42] (2019). Plywood—Bonding Quality—Part 1: Test Methods.

