# Supplementary material for: Polyelectrolyte Complex/MMT Hybrid for SPI/WE Adhesives with Balanced Flame Retardancy and Shear Strength
Source: Gels. 2026 Sep 11;12(9):834. doi: 10.3390/gels12090834 (PMC13606767; doi:10.3390/gels12090834)
Supplement: Supplementary file 1 [file gels-12-00834-s001.zip › gels-4517321-supplementary.pdf]

Supporting Information

Polyelectrolyte Complex/MMT Hybrid for SPI/WE Adhesives with Balanced Flame Retardancy and Shear Strength

Han Yang [1,2,*], Yuxin Luo [3], Shiyi Ding [2], Chunchun Wu [1] and Tao Zhang [4,*]

[1] School of Materials Science and Engineering, Zhejiang University, Hangzhou 310058, China; wuchun@zju.edu.cn
[2] Zhejiang Provincial Fire and Rescue Bureau, Hangzhou 310008, China; srsrsre@163.com
[3] School of Materials Science & Engineering, Zhejiang Sci-Tech University, Hangzhou 310018, China; yamy_luo@163.com
[4] Department of Product Design, School of Art & Design, Zhejiang Sci-Tech University, Hangzhou 310018, China
* Correspondence: 12126093@zju.edu.cn (H.Y.); taoyuan0510@126.com (T.Z.)

Contents

Table S1-S3
Figures S1-S4

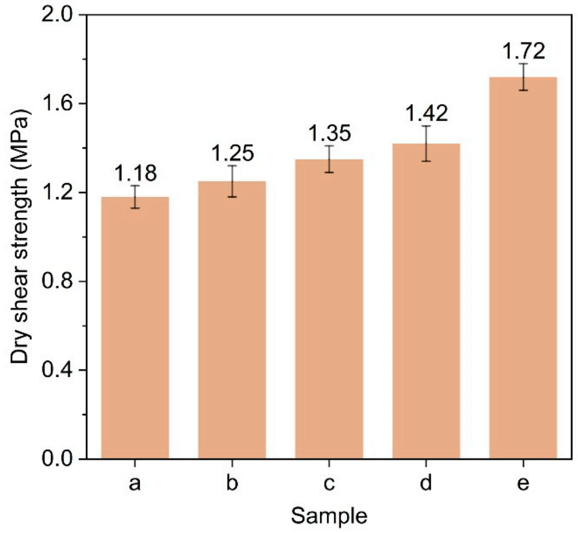

Figure S1. Dry shear strength values of (a) SPI, (b) SPI/5WE, (c) SPI/10WE, (d) SPI/20WE and (e) SPI/40WE. Error bars represent standard deviation (SD).

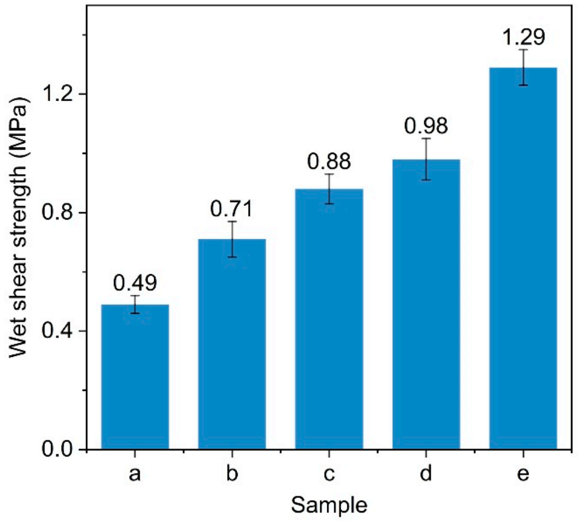

Figure S2. Wet shear strength values of (a) SPI, (b) SPI/5WE, (c) SPI/10WE, (d) SPI/20WE and (e) SPI/40WE. Error bars represent standard deviation (SD).

Table S1. Adhesive formulations of SPI/WE/THPS@CS/MMT.

Sample	Proportions
SPI	*M* (SPI)/*M* (water)=30:70
SPI/20WE	*M* (SPI)/*M* (EPOXY) /*M* (curing agent) /*M* (water)=30:6:3:70
SPI/20WE/THPS	*M* (SPI)/*M* (EPOXY) /*M* (curing agent) /*M* (THPS)/*M* (water)=30:6: 3: 0.45:70
SPI/20WE/CS	*M* (SPI)/*M* (EPOXY)/ *M* (curing agent) *M* (CS)/*M* (water)=30:6: 3: 1.35:70
SPI/20WE/THPS@CS	*M* (SPI)/*M* (EPOXY) / *M* (curing agent) /*M* (CS)/*M* (THPS)/*M* (water)=30:6: 3: 1.35:0.45:70
SPI/20WE/THPS@CS/MMT	*M* (SPI)/M (EPOXY) / *M* (curing agent) /*M* (MMT)/*M* (CS)/*M* (THPS)/*M* (water)=30:6: 3: 0.45:1.35:0.45:70

Note: THPS@CS was prepared with a THPS:CS solid mass ratio of 1:3 (see Section 4.2 for details). The WE content throughout this work is defined as the mass ratio of pure WE solid resin to the solid mass of SPI; the corresponding commercial WE dispersion and its curing agent were added based on their solid contents to achieve the desired solid loading; the curing agent is added at a fixed solid mass ratio of WE: curing agent = 2:1 and is not included in the WE dosage calculation.

Table S2. Thermal degradation behaviors of CS and THPS@CS under nitrogen condition.

Sample	$T_{5\%}$ (°C)	T_{max} (°C)	MLR_{max} (wt%/ °C)	Char at 600 °C (wt %)
CS	280	302	0.91	42.4
THPS@CS	207	220	0.64	49.2

Table S3. Elemental compositions of SPI, SPI/20WE, SPI/20WE/THPS@CS and SPI/20WE/THPS@CS/MMT.

Sample	C (%)	N (%)	O (%)	P (%)	S (%)	Si (%)
SPI	72.2	5.0	22.8	--	--	--
SPI/20WE	76.5	3.6	19.9	--	--	--
SPI/20WE/THPS@CS	83.0	1.2	15.0	0.7	0.1	--
SPI/20WE/THPS@CS/MMT	80.1	1.3	16.2	1.1	0.2	1.1

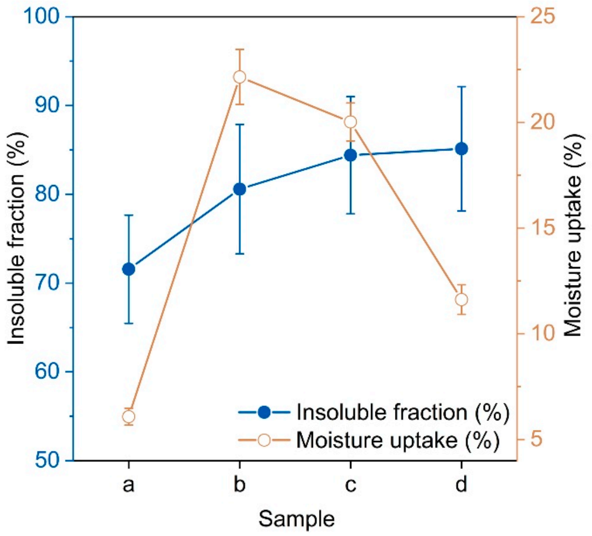

Figure S3. Insoluble fraction and moisture uptake values of (a) SPI, (b) SPI/20WE, (c) SPI/20WE/THPS@CS and (d) SPI/20WE/THPS@CS/MMT. Error bars represent standard deviation (SD).

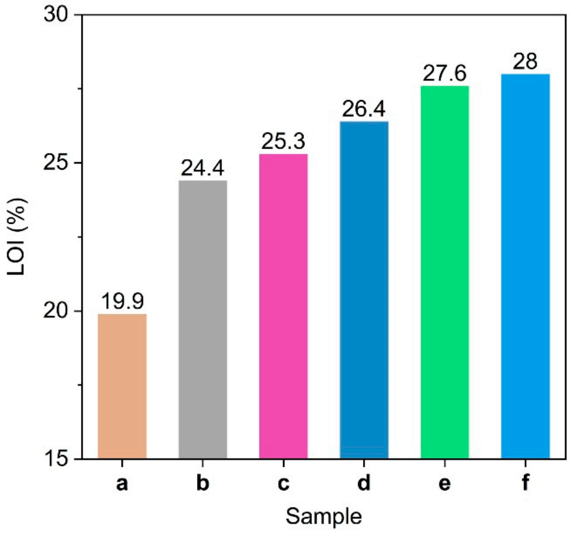

Figure S4. LOI values of (a) untreated wood, and the two-ply plywood (130 mm × 65 mm × 4 mm) bonded with (b) SPI, (c) SPI/20WE, (d) SPI/20WE/CS, (e) SPI/20WE/THPS@CS and (f) SPI/20WE/THPS@CS/MMT.
